# Dynamic LTR retrotransposon transcriptome landscape in septic shock patients

**DOI:** 10.1186/s13054-020-2788-8

**Published:** 2020-03-18

**Authors:** Marine Mommert, Olivier Tabone, Audrey Guichard, Guy Oriol, Elisabeth Cerrato, Mélanie Denizot, Valérie Cheynet, Alexandre Pachot, Alain Lepape, Guillaume Monneret, Fabienne Venet, Karen Brengel-Pesce, Julien Textoris, François Mallet, MIPrea Study Group, Christine Alberti-Segui, Bernard Allaouchiche, Laurent Argaud, Frédéric Aubrun, Véronique Barbalat, Thomas Baudry, Julien Bohé, Marie-Angélique Cazalis, Elisabeth Cerrato, Martin Cour, Sylvie De La Salle, Benjamin Delwarde, Bernard Floccard, Arnaud Friggeri, Emmanuelle Gallet-Gorius, Christian Guillaume, Romain Hernu, Audrey Larue-Triolet, Alain Lepape, Guillaume Marcotte, Delphine Maucort-Boulch, Boris Meunier, Guillaume Monneret, Stéphane Morisset, Julie Mouillaux, Alexandre Pachot, Mathieu Page, Nathalie Panel, Estelle Peronnet, Vincent Piriou, Anne Portier, Marion Provent, Thomas Rimmelé, Marie Simon, Julien Textoris, Fabrice Thiolliere, Hélène Vallin, Fabienne Venet, REALISM Study Group, André Boibieux, Julien Davidson, Laure Fayolle-PivoT, Julie Gatel, Charline Genin, Arnaud Gregoire, Alain Lepape, Anne-Claire Lukaszewicz, Guillaume Marcotte, Marie Matray, Delphine Maucort-Boulch, Guillaume Monneret, Nathalie Panel, Thomas Rimmele, Hélène Vallin, Fabienne Venet, Sophie Blein, Karen Brengel-Pesce, Elisabeth Cerrato, Valérie Cheynet, Emmanuelle Gallet-Gorius, Audrey Guichard, Camille Jourdan, Natacha Koenig, François Mallet, Boris Meunier, Virginie Moucadel, Marine Mommert, Guy Oriol, Alexandre Pachot, Claire Schrevel, Olivier Tabone, Julien Textoris, Javier Yugueros Marcos, Jérémie Becker, Frédéric Bequet, Yacine Bounab, Florian Brajon, Bertrand Canard, Muriel Collus, Nathalie Garcon, Irène Gorse, Cyril Guyard, Fabien Lavocat, Philippe Leissner, Karen Louis, Maxime Mistretta, Jeanne Moriniere, Yoann MouscaZ, Laura Noailles, Magali Perret, Frédéric Reynier, Cindy Riffaud, Mary-Luz Rol, Nicolas Sapay, Trang Tran, Christophe Vedrine, Christophe Carre, Pierre Cortez, Aymeric De Monfort, Karine Florin, Laurent Fraisse, Isabelle Fugier, Maïna L’Azou, Sandrine Payrard, Annick PELERAUX, Laurence Quemeneur, Andrew Griffiths, Stephanie Toetsch, Teri Ashton, Peter J. Gough, Scott B. Berger, David Gardiner, Iain Gillespie, Aidan Macnamara, Aparna Raychaudhuri, Rob Smylie, Lionel Tan, Craig Tipple

**Affiliations:** 1 Joint Research Unit, bioMerieux, Centre Hospitalier Lyon Sud, Hospice Civils de Lyon, 165 Chemin du Grand Revoyet, 69310 Pierre-Benite, France; 2 EA 7426 Pathophysiology of Injury-Induced Immunosuppression, University of Lyon1-Hospices Civils de Lyon-bioMérieux, Hôspital Edouard Herriot, 5 Place d’Arsonval, 69437 Lyon Cedex 3, France; 3 Intensive Care Unit, Centre Hospitalier Lyon Sud, Hospices Civils de Lyon, Pierre Bénite, France; 4 Emerging Pathogens Laboratory, Epidemiology and International Health, International Center for Infectiology Research (CIRI), Lyon, France; 5 bioMérieux Joint Research Unit, Hospices Civils de Lyon, Groupement Hospitalier Edouard Herriot, Lyon, France; 6 Immunology Laboratory, Hospices Civils de Lyon, Groupement Hospitalier Edouard Herriot, Lyon, France; 7 Department of Anaesthesiology and Critical Care Medicine, Hospices Civils de Lyon, Groupement Hospitalier Edouard Herriot, Université Claude Bernard Lyon 1, Lyon, France

**Keywords:** HERV transcriptome, Whole blood, Septic shock patients, mHLA-DR expression, Immunosuppression

## Abstract

**Background:**

Sepsis is defined as a life-threatening organ dysfunction caused by a dysregulated host response to infection. Numerous studies have explored the complex and dynamic transcriptome modulations observed in sepsis patients, but a large fraction of the transcriptome remains unexplored. This fraction could provide information to better understand sepsis pathophysiology. Multiple levels of interaction between human endogenous retroviruses (HERV) and the immune response have led us to hypothesize that sepsis is associated with HERV transcription and that HERVs may contribute to a signature among septic patients allowing stratification and personalized management.

**Methods:**

We used a high-density microarray and RT-qPCR to evaluate the HERV and Mammalian Apparent Long Terminal Repeat retrotransposons (MaLR) transcriptome in a pilot study that included 20 selected septic shock patients, stratified on mHLA-DR expression, with samples collected on day 1 and day 3 after inclusion. We validated the results in an unselected, independent cohort that included 100 septic shock patients on day 3 after inclusion. We compared septic shock patients, according to their immune status, to describe the transcriptional HERV/MaLR and conventional gene expression. For differential expression analyses, moderated *t* tests were performed and Wilcoxon signed-rank tests were used to analyze RT-qPCR results.

**Results:**

We showed that 6.9% of the HERV/MaLR repertoire was transcribed in the whole blood, and septic shock was associated with an early modulation of a few thousand of these loci, in comparison to healthy volunteers. We provided evidence that a subset of HERV/MaLR and conventional genes were differentially expressed in septic shock patients, according to their immune status, using monocyte HLA-DR (mHLA-DR) expression as a proxy. A group of 193 differentially expressed HERV/MaLR probesets, tested in an independent septic shock cohort, identified two groups of patients with different immune status and severity features.

**Conclusion:**

We demonstrated that a large, unexplored part of our genome, which codes for HERV/MaLR, may be linked to the host immune response. The identified set of HERV/MaLR probesets should be evaluated on a large scale to assess the relevance of these loci in the stratification of septic shock patients. This may help to address the heterogeneity of these patients.

## Background

Sepsis is defined as a life-threatening organ dysfunction caused by a dysregulated host response to infection, with septic shock being the most severe subtype of sepsis [1]. Despite major progress, due in large part to source control with antibiotics and early initiation of intensive care therapy, sepsis remains a major health issue, with a high worldwide prevalence and high mortality rate. It is characterized by immune dysfunctions with concomitant excessive pro- and anti-inflammatory responses, which can lead to organ failure, immunoparalysis, and secondary infections. The pathophysiological mechanisms of sepsis are not completely understood. The early model of an overwhelming inflammatory reaction failed to capture the complex pathophysiology of the syndrome, as proven by the failure of multiple clinical trials with a variety of anti-inflammatory agents [2]. While an immune dysregulated host response is clearly part of the pathophysiology, the complex interactions between the immune response and other key physiological systems, such as the autonomous nervous system, coagulation, or cellular bioenergetics, are still poorly understood [3, 4]. This translates into an apparent heterogeneity observed in patients, which makes the selection of appropriate therapeutic care a major clinical challenge. Several markers of the immune status have been explored to better stratify patients [5]. There are currently no gold standard or clinical signs to evaluate immunosuppression in the intensive care unit. To date, HLA-DR expression in monocytes, measured by flow cytometry, is a well-accepted and beneficial marker for monitoring immune alterations in critically ill patients (sepsis, trauma, pancreatitis, surgery, and burns). The decreased expression of mHLA-DR has been repeatedly associated with mortality and secondary infections and therefore remains an independent predictor of a poor outcome after sepsis [4, 6–10].

Long terminal repeat (LTR) retrotransposons, which represent 8.3% of the human genome [11] were recently hypothesized to be relevant in the pathophysiology of sepsis [12, 13]. These retrovirus-like sequences consist of approximately 200,000 human endogenous retroviruses (HERVs) and 240,000 mammalian apparent long terminal repeat retrotransposons (MaLR). The expression of HERVs has been observed in both inflammation [14, 15] and immunosuppression [13, 16], and the insertional polymorphism of HERV LTRs at the HLA locus has been associated with several auto-immune diseases [17]. In addition, several lines of evidence support the role of HERVs as contributors to the immune response [18]. For example, loss of DNA methylation triggers cytosolic sensing of double-stranded RNA (dsRNA) and overexpression of HERVs, both of which cause a type I interferon response and apoptosis [19]. Another study in cancer highlighted that overexpression of HERVs triggers pathologic innate immune signaling [20]. Chuong et al. also showed that HERV elements constitute a dynamic reservoir of IFN-inducible enhancers that allow the regulation of essential immune functions [21]. These data led us to hypothesize that sepsis may be associated with HERV transcription, which may modulate the immune response. Moreover, irrespective of causal or consecutive expression, HERVs could be part of an informative molecular signature that may allow a better stratification of septic shock patients and improved patient management.

**Fig. 1:**
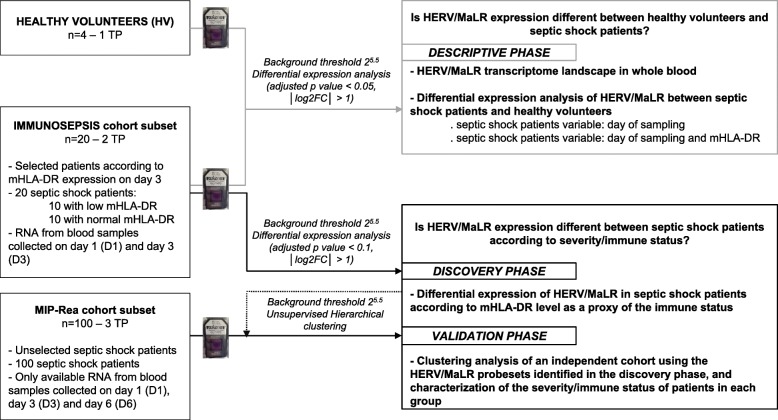
Schematic representation of the data process analysis. Healthy volunteers and both septic shock patient cohort subsets (IMMUNOSEPSIS and MIP-Rea) are defined on the left. Bioinformatics analysis parameters used for each step are described in the middle. Finally, the three phases of the data process analysis (descriptive phase, discovery phase, and validation phase) are presented. TP time point, D day

We have recently shown that several HERVs are expressed and modulated after septic shock and other inflammatory injuries [12]. Using the HERV-V3 microarray, which allows the measurement of HERV/MaLR at the individual locus level [22], we have also shown that HERV/MaLR are tightly modulated in peripheral blood mononuclear cells (PBMCs). We have used an ex vivo endotoxin tolerance model to mimic the septic shock host immune response [13]. The data from this pilot study were used to investigate the HERV/MaLR transcriptome in septic shock patients, who were stratified by monocyte HLA-DR (mHLA-DR) expression on day 3, following intensive care unit (ICU) admission (Fig. 1). We provided a global view of the HERV transcriptome in the whole blood of healthy volunteers and septic shock patients. We also aimed to identify whether a subset of HERV/MaLR was expressed in septic shock patients, according to their immune status, as estimated by mHLA-DR expression. Finally, we highlighted a group of 193 differentially expressed HERV/MaLR probesets, which identified two groups of patients with different immune status and severity features, in an independent septic shock cohort.

## Methods

### Biological samples

#### IMMUNOSEPSIS cohort subset

We retrospectively selected patients from a prospective, non-interventional study conducted in Lyon, France, named IMMUNOSEPSIS. A cohort of adult septic shock patients was enrolled from December 2001 to April 2005 in two ICUs from a French university hospital. The clinical study was approved by the regional ethics committee (Comité Consultatif de Protection des Personnes dans la Recherche BioMédicale de Lyon A). The committee waived the need for written informed consent because the study was observational, with a low risk to patients, and no specific procedure, other than routine blood sampling, was required. Oral information and non-opposition to inclusion in the study were mandatory and were systematically obtained before any blood sample was drawn. This was recorded in patients’ clinical files. The cohort has been described in detail elsewhere [23, 24]. In brief, blood samples were collected in PAXgene™ Blood RNA tubes (PreAnalytix) and RNA was extracted and frozen at − 80 °C, as previously described [25]. For the current study, we selected 20 patients for which the mHLA-DR expression level was measured on the monocyte cell surface by flow cytometry from the peripheral whole blood collected in EDTA anticoagulant tubes, as previously described [26, 27]. We chose 10 patients with a normal expression level of mHLA-DR on day 3 (more than 30% of expression on monocytes cell surface) and 10 patients with a low expression level of mHLA-DR on day 3 (less than 30% of expression on monocytes cell surface), for which RNA samples were available on day 1 and day 3 or 4 (to simplify, named day 3, thereafter). The RNA integrity was measured prior to RNA amplification using a Bioanalyser 2100 (Agilent), in accordance with the manufacturer’s instructions (median RNA integrity number 8.5 [min 6.5–max 9.4]). Patient characteristics of the IMMUNOSEPSIS cohort subset are described in Table 1.

**Table 1:** Patient characteristics of the IMMUNOSEPSIS cohort subset according to the mHLA-DR expression on day 3 (descriptive and discovery phase)

Variable	Normal HLA DR on day 3 (*n* = 10)	Low HLA DR on day 3 (*n* = 10)	Whole cohort (*n* = 20)	*P* value
Demographics				
Gender (male)	8 (80)	5 (50)	13 (65)	0.315
Age (years)	59 [54–67]	57 [46–69]	59 [52–68]	0.665
Admission data				
SOFA Score day 1	8 [6–9]	11 [9–16]	9 [8–12]	**0.018**
SAPSII day 1	44 [33–45]	58 [47–68]	45 [34–56]	0.052
Primary site of infection				0.912
Pulmonary	4 (20)	4 (20)	8 (40)	
Abdominal	3 (15)	5 (25)	8 (40)	
Other	3 (15)	1 (5)	4 (20)	
Type of primary infection				0.631
Community acquired	3 (15)	6 (30)	9 (45)	
Hospital acquired	6 (30)	3 (15)	9 (45)	
Reanimation acquired	1 (5)	1 (5)	2 (10)	
Type of detection				
Microbiologically documented infection	0 (0)	2 (10)	2 (10)	0.474
Primary site of infection	10 (50)	8 (40)	18 (90)	0.474
Hydrocortisone	5 (25)	7 (35)	12 (60)	0.739
Monocyte HLA-DR expression				
% of mHLA-DR on day 1	63 [50–70]	29 [20–42]	45 [31–70]	**0.007**
% of mHLA-DR on day 3	63 [51–80]	17 [12–25]	36 [18–63]	**< 0.001**
Outcomes				
Secondary infection	0 (0)	2 (20)	2 (10)	0.796
Hospital length of stay (days)	38.5 [28–58]	31 [14–42]	34 [21–48]	1.000
ICU length of stay (days)	5.5 [4–20]	8 [5–10]	6.5 [5–13]	1.000
Non-survivors at day 28	2 (20)	7 (70)	9 (45)	0.052

Categorical variables are expressed as *n*(%) and continuous variables as median [Q1–Q3]. Comparisons between normal and low mHLA-DR expression groups, at day 3, were performed with a Chi-squared test for qualitative variables and Mann-Whitney test for quantitative variables, as appropriate. Values in bold indicate significance at *p* < 0.05. *ICU* intensive care unit, *SOFA* sequential organ failure assessment, *SAPS* Simplified Acute Physiology Score

#### Healthy controls

PAXgene™ Blood RNA tubes (PreAnalytix) from four healthy volunteers (HV), matched in age (median at 52 years [Q1:51–Q3:54]) and sex (75% were male), were independently obtained from EFS (Etablissement Français du Sang) and used immediately. We used the EFS standardized procedures for blood donation and followed provisions of articles R.1243–49 and the French public health code to obtain written non-opposition to the use of donated blood for research purposes from HV. The blood donors’ personal data were deidentified before transfer to our research laboratory. We obtained the favorable notice of the local ethical committee (Comité de Protection des Personnes Sud-Est II, Bâtiment Pinel, 59 Boulevard Pinel, 69,500 Bron) and acceptance from the French ministry of research (Ministère de lʼEnseignement supérieur, de la Recherche et de lʼInnovation, DC-2008-64) for the handling and conservation of these samples. Blood samples were stabilized for at least 4 h at room temperature, after collection, and frozen at − 80 °C, following the manufacturer’s guidelines. Total RNA was extracted using the PAXgene Blood RNA kit (PreAnalytix), in accordance with the manufacturer’s instructions. The RNA quantity and quality were determined using a Nanodrop (Thermo Scientific) Bioanalyser 2100 (Agilent), in accordance with the manufacturer’s instructions. Samples with an RNA integrity number ≤ 6 were excluded due to poor quality RNA.

#### MIP-Rea cohort subset

We retrospectively selected patients from a prospective, multicenter, non-interventional study, named MIP-Rea, that was conducted in six ICUs in Lyon, France. The study was approved by our institutional ethical review board (Comité d’Ethique des Centres d’investigation Clinique de l’Inter-Région Rhône-Alpes Auvergne – IRB 5044), and consent for an ancillary study was obtained a posteriori. The protocol of this study was submitted to the French CCTIRS and CNIL committees and approved on April 22, 2016, and September 30, 2016, respectively. Further, informed consent was received from patients for inclusion in this specific study. The cohort has been described in detail elsewhere [28]. In brief, blood samples were collected in PAXgene™ Blood RNA tubes (PreAnalytix) and RNA was extracted and frozen at − 80 °C, as previously described [28]. For the current study, we selected only septic shock patients for which RNA samples were available on day 1, day 3, and day 6 (corresponding to a total of 100 septic shock patients). The RNA integrity was measured prior to RNA amplification using a Bioanalyser 2100 (Agilent), in accordance with the manufacturer’s instructions (median RNA integrity number 7.9 [min 6–max 9.6]). Patient characteristics for the MIP-Rea cohort subset are described in Table 2.

**Table 2:** Patient characteristics of the MIP-Rea cohort subset according to cluster affiliation described in the “Results” section (validation phase)

Variable	Cluster 1 (*n* = 60)	Cluster 2 (*n* = 40)	Total (*n* = 100)	*P* value
Demographics				
Gender (male)	40 (67)	27 (68)	67 (67)	1.000
Age (years)	71 [58–77]	67 [61–76]	70 [59–77]	0.830
Admission data				
SAPSII	62 [52–74]	66 [48–72]	63 [51–73]	0.530
Glasgow score	14 [9–15]	14 [8–15]	14 [8–15]	0.167
SOFA Score day 1	12 [10–14]	10 [8–12]	12 [9–14]	0.103
SOFA Score day 3	11 [8–14]	8 [6–11.00]	9 [7–13]	**0.003**
SOFA Score day 6	8 [5–11]	5 [3–7]	6 [4–10]	**< 0.001**
Primary site of infection				0.735
Pulmonary	31 (52)	24 (60)	55 (55)	
Abdominal	14 (23)	7 (18)	21 (21)	
Other	15 (25)	9 (23)	24 (24)	
Type of primary infection				1.000
Community acquired	40 (67)	26 (65)	66 (66)	
Hosrpital acquired	20 (33)	14 (35)	34 (34)	
Type of detection				0.061
Clinical+imaging	9 (15)	8 (20)	17 (17)	
Clinical+surgery	2 (3)	3 (8)	5 (5)	
Microbiology	48 (80)	24 (60)	72 (72)	
Suspected	1 (2)	5 (13)	6 (6)	
Hydrocortisone	47 (81)	21 (53)	68 (68)	**0.005**
Hematology on day 3				
White cells (10^9^/L)	(*n* = 60)	(*n* = 39)	(*n* = 99)	**< 0.001**
	16.3 [11.8–21.1]	11 [8.6–15.6]	14.7 [9.9–18.7]	
Neutrophils (10^9^/L)	(*n* = 47)	(*n* = 30)	(*n* = 77)	**< 0.001**
	15.4 [11.1–19.7]	8.9 [7–13.2]	13 [8.6–17.2]	
Lymphocytes (10^9^/L)	(*n* = 47)	(*n* = 30)	(*n* = 77)	**0.001**
	0.7 [0.4–0.8]	1 [0.7–1.1]	0.8 [0.6–1]	
Platelets (10^9^ /L)	(*n* = 60)	(*n* = 39)	(*n* = 99)	**< 0.001**
	94.5 [54.8–147.3]	170 [105–242.5]	119 [72–200]	
Molecular markers				
CD74 ratio (D3/D1; CNRQ)	0.97 [0.59–1.34]	1.60 [1.03–1.96]	1.21 [0.68–1.69]	**< 0.001**
HLA-DRA day 3 (CNRQ)	0.20 [0.14–0.30]	0.50 [0.37–0.79]	0.31 [0.18–0.55]	**< 0.001**
CX3CR1 day 3 (CNRQ)	0.13 [0.08–0.22]	0.42 [0.28–0.65]	0.21 [0.10–0.39]	**< 0.001**
S100A9 day 3 (CNRQ)	11.34 [7.28–15.58]	6.47 [5.05–8.66]	8.80 [6.03–13.26]	**< 0.001**
Outcomes				
Secondary infection	16 (16)	10 (10)	26 (26)	0.852
Hospital length of stay (days)	25 [17–48]	35 [20–56]	29 [18–54]	0.241
ICU length of stay (days)	15 [10–20]	13 [9–22]	14 [10–21]	0.319
Non survivors on day 28	16 (27)	7 (18)	23 (23)	0.410

Categorical variables are expressed as *n*(%) and continuous variables as median [Q1–Q3]. Comparisons between two clusters were performed with the chi-squared test for qualitative variables and Mann-Whitney test for quantitative variables, as appropriate. Values in bold indicate significance at *P* < 0.05. *ICU* intensive care unit, *SOFA* Sequential Organ Failure Assessment, *SAPS* Simplified Acute Physiology Score, *D* day, *CNRQ* Calibrated Normalized Relative Quantities

### HERV-V3 processing and analysis

#### Custom Affymetrix HERV-V3 GeneChip microarray

The HERV-V3 microarray targeted 353,994 loci-elements, which were represented by 4,410,200 probes [22]. The custom HERV GeneChip can discriminate distinct HERV elements through the use of a set of highly informative probesets (located in U3, R, U5 subdomains of solo, 5′ and 3′ individual LTRs and *gag*/*pol*/*env* regions), hereafter named the “HERV prototypes repertoire.” A group of probesets with lower quality annotations (located in the first third and last third of the complete LTR and every 2.5 kb in the region in between the LTRs) was also used and is hereafter referred to as the “HERV/MaLR_Dfam repertoire.” The custom HERV GeneChip also contained probesets that target 1500 genes involved in the immune response. The description of the HERVgDB4 database and the final content of the HERV-V3 microarray are provided in Additional file 1: Table S1 and were previously described in Becker et al. [22].

#### RNA amplification, labeling, and HERV-V3 microarray hybridization

The cDNA synthesis and amplification steps were performed from 16 ng of RNA, using the Ovation Pico WTA System V2 kit (Nugen), in accordance with the manufacturer’s instructions. Five micrograms of purified, amplified cDNA was fragmented into 50–200 bp fragments and were 3′-labeled using the Encore Biotin Module kit (Nugen), in accordance with the manufacturer’s instructions. The HERV-V3 microarrays were hybridized at 50 °C for 18 h in an oven, with constant stirring (60 rpm). Washing and staining were performed using the protocol supplied by the manufacturer, using the GeneChip fluidic station 450 (Affymetrix). The arrays were scanned using the GeneChip fluorometric scanner 3000 7G (Affymetrix). Images (DAT files) were converted to CEL files using the GCOS software (Affymetrix) [22]. The experimental data generated were deposited in the National Center for Biotechnology Information (NCBI) and are available in the GEO DataSets site, under accession number GSE121352.

#### Microarray analysis preprocessing

The CEL files were transformed into a matrix, then normalized and adjusted for background noise (RMA normalization). Probes were summarized into probesets with command apt-probeset-summarize (v1.18.0). Microarray preprocessing and statistical analyses were performed using R/Bioconductor (R v3.1.2) [29]. Quality assessment was performed through simpleaffy (v2.42.0) [30] and arrayQualityMetrics (v3.22.1) [31]. For quality control, several criteria were used: RNA quality, images of chips, hybridization spike-in, polyA, amplification and fragmentation, intensity signals (before and after normalization), probeset homogeneity (RLE, NUSE plots), correlation plots (before and after normalization), and Principal Component Analysis. For most criteria, outlier detection was performed by computing the Kolmogorov Smirnof (KS) statistic between each array and the pooled data (default threshold with arrayQualityMetrics library). One array, which corresponded to a HV sample, was removed from the analysis as it did not pass more than four quality controls. This left four HV samples for the analysis. Experiment batch effects were removed using COMBAT [32]. Finally, low expression probesets were filtered to reduce the dataset size and gain statistical power for the analyses. A coefficient of variation (CV) value of 10% was used to determine the intensity threshold (2^5.5^) to filter such probesets with low expression. Probesets under this intensity threshold in more than 90% of all samples (40 samples out of the 44) were removed, which left 120,222 probesets for the analysis. LTR function analysis and RT-qPCR validation not described in the following section are presented in the Additional file 2: Supplementary Methods.

#### Bioinformatics analysis

For differential expression analyses, moderated *t* tests were performed (Limma (v3.22.7) R package) [33], and *P* values were adjusted for multiple testing using the Benjamini-Hochberg procedure [34]. When we compared septic shock patients and HV, we used absolute log_2_ Fold Change (|log_2_FC|) higher than 1 and an adjusted *P* value lower than 0.05. When we compared septic shock patient groups, according to the day of sampling and mHLA-DR, a probeset was considered to show statistically significant differential expression when the |log_2_FC| was higher than 1 and adjusted *P* value was lower than 0.1. Graphics were drawn using ggplot2 (v2.2.0) or pheatmap (v1.0.8). The principal component analysis was performed on the HERV expression matrix. The representation on the first two components was carried out using the ggplot2 library. The two vectors drawn on the plot are the median of coordinate differences between day 1 and day 3 for each patient, according to their mHLA-DR status. Finally, the 193 HERV/MaLR probesets that were modulated on day 3, between low and normal mHLA-DR patients from the discovery phase, were tested on the independent cohort using unsupervised hierarchical clustering. The complete clustering method was applied, with Euclidean distance for rows (probesets) and Pearson’s correlation distance for columns (samples).

## Results

### HERV/MaLR transcriptome landscape in the whole blood

**Fig. 2:**
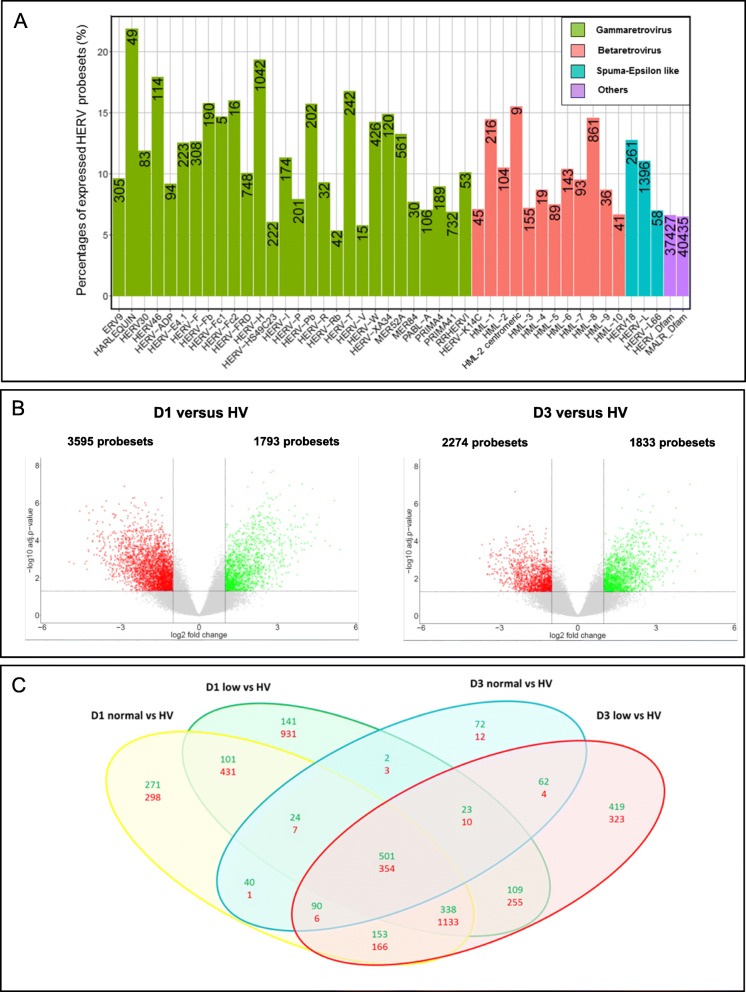
HERV/MaLR transcriptome landscape in the whole blood (descriptive phase). **a** Percentages and absolute counts (number presented inside the bars) of expressed loci (probeset intensity above threshold), within individual groups of “HERV_prototypes,” “HERV_Dfam,” and “MaLR_Dfam” repertoires. “HERV_prototypes” were grouped by classes of retroviruses, namely gamma-retrovirus (green), beta-retrovirus (pink), and spuma-epsilon like retrovirus (blue). “HERV and MaLR Dfam” repertoires are depicted as a global homogeneous entity (purple). **b** Volcano plots derived from the HERV/MaLR differential expression analysis, between septic shock patients and HV on day 1 (left) or day 3 (right). The *x*-axis represents the log_2_ fold change values and the *y*-axis represents the -log_10_ adjusted *P* values. Each point represents a given probeset. Statistically and biologically significant probesets (adjusted *P* value < 0.05, │log_2_FC│ > 1) are colored (red = downregulated, green = upregulated). **c** Venn diagrams from the HERV/MaLR differential expression analyses, according to the day of sampling and mHLA-DR expression (compared to HV). Upregulated probesets are in green and downregulated probesets are in red

We assessed the HERV/MaLR transcriptome from whole blood in a selected IMMUNOSEPSIS cohort subset [23, 24] that consisted of 20 septic shock patients (samples collected at admission and on day 3) and four HV (named descriptive phase in Fig. 1). A summary of the absolute counts and the relative abundances of transcriptionally active elements of the HERV/MaLR transcriptome are given in Fig. 2a (and extended in Additional file 1: Table S1). Overall, 6.9% (87,912 probesets) of HERV/MaLR were transcriptionally active in the whole blood, which was relatively low compared to the observed percentage of expressed genes (42%) among the set of 1500 immune-related genes (32,310 probesets). All well-defined HERV groups had expressed loci in the whole blood, and we did not observe significant enrichment or depletion of a specific repertoire^1^, class^2^, or HERV group^3^, between HV and septic shock patients (data not shown). Among the expressed prototype elements, 7.4% belonged to gamma-retroviruses and were mostly found in the HERV-H group (1042 expressed probesets, which corresponded to 19% of the whole group). It should be noted that the HERV-FRD and PRIMA41 groups contained a high absolute number of expressed loci (748 and 732 probesets, respectively). In beta-retroviridae, 2.1% of elements were expressed, mostly from the HML-8 and HML-1 groups (861 probesets, 14% of the whole group; and 216 probesets, 14% of the whole group, respectively). Finally, 2% of the expressed elements belonged to spuma/epsilon-like retrovirus classes, in which the HERV-L group provided the largest count of expressed probesets (1396 probesets, which corresponded to 11% of the whole group). The overall HERV/MaLR transcriptome analysis from the whole blood was also carried out on 100 septic shock patients from the independent and unselected MIP-REA cohort subset [28] (named validation phase in Fig. 1), and the results were similar (Additional file 3: Figure S1). Interestingly, 92% of expressed probesets were common to both cohorts.

Beyond the overall transcription analysis, we looked for any difference due to sequence composition (LTRs^4^, versus proviral genes) or function (promoter versus polyA). In Additional file 4: Figure S2, we summarized the HERV/MaLR transcriptomic activity and LTR putative regulatory functions observed in HV and patients. We found enrichment in expressed LTRs when compared to the expressed proviral regions of HERVs. We also found that 22% of LTRs were either putative promoters (Pr) or polyadenylation (pA) signals. About one third of LTRs shifted from a silent to active state (Pr or pA) between samples used in the descriptive phase. Notably, the LTR switch of function from promoter to polyA appeared to be extremely rare. This supported our previous assumption that an LTR is predetermined to act as a promoter or a polyA signal, which is a phenomenon that we named “operational determinism” [13, 35]. Overall, these results confirmed, in septic shock patients, what was previously observed in an endotoxin tolerance model in PBMC in vitro [13]*.*

We then performed a differential expression analysis between the HV and septic shock patients on either day 1 or day 3. The exhaustive dataset of this analysis is presented in Additional file 5: Table S2. Overall, we found 855 differentially expressed HERV/MaLR probesets between combined patient samples (integrating day of sampling and mHLA-DR status) and HV. Volcano plots were depicted at the probeset level for HERV/MaLR (Fig. 2b). There were approximately twice as many downregulated as upregulated HERV/MaLR, when comparing septic shock patients on day 1 with HV (513 upregulated probesets vs 1660 downregulated probesets). The difference between the two groups appeared to be smaller on day 3 (553 upregulated probesets vs 339 downregulated probesets) (Fig. 2b, c. When mHLA-DR expression was added as a variable, the overall number of downregulated HERV/MaLR probesets was more higher in septic shock patients with low mHLA-DR expression versus normal mHLA-DR expression compared to HV on day 1 (931 downregulated probesets vs 298 upregulated probesets) but also on day 3 (323 downregulated probesets vs 12 upregulated probesets) (Fig. 2c and Additional file 5: Table S2). Globally, we confirmed that septic shock is associated with an early specific modulation of the expression of a few thousands loci from the LTR retrotransposon repertoire of the human genome.

### The differential expression of HERV/MaLR according to immune status of septic shock patients

**Fig. 3:**
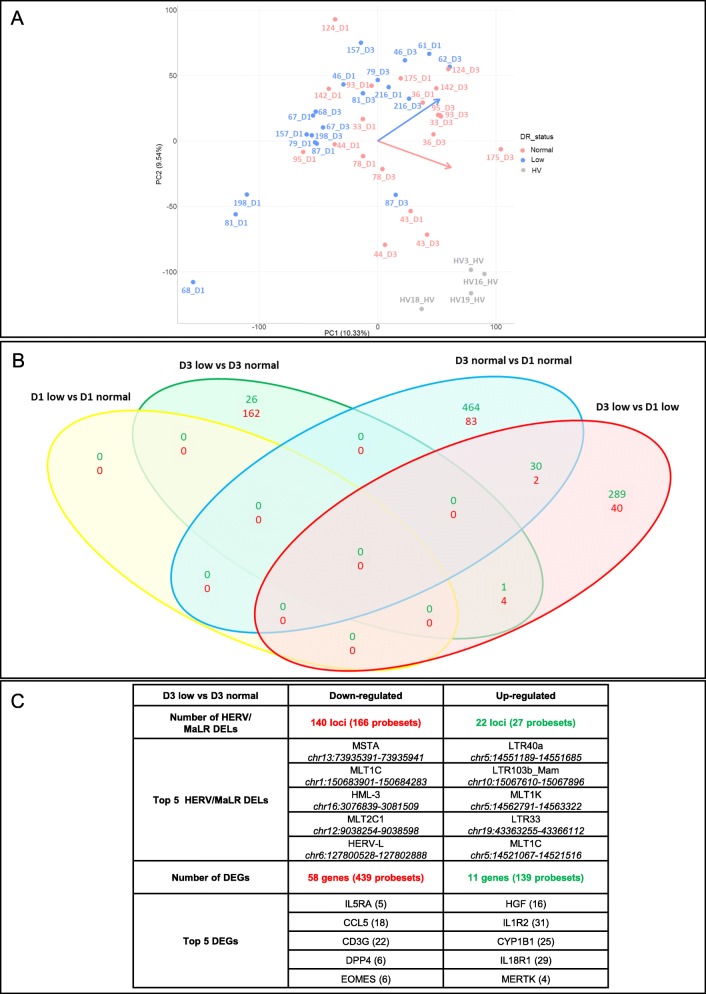
The differential expression of HERV/MaLR according to the immune status of septic shock patients (discovery phase). **a** Principal component analysis developed from the HERV expression matrix. Healthy volunteers, patients with normal expression of mHLA-DR, and patients with low expression of mHLA-DR are indicated in gray, pink, and blue, respectively. **b** Venn diagrams from HERV/MaLR differential expression analyses, according to the day of sampling and mHLA-DR expression (compared between septic shock patient groups). Upregulated probesets are in green and downregulated probesets are in red. **c** The table shows the number of statistically significant differentially expressed loci (DELs) for HERV/MaLR, differentially expressed genes (DEGs), and the corresponding number of probesets, respectively. Downregulated loci are in red, upregulated loci are in green. For HERV/MaLR loci, the name, number of differentially expressed probesets (between brackets), and chromosomal locations (in italic) are indicated (GRCh38 genome version) for the five most differentially expressed loci. For genes, the official gene symbol and the number of differentially expressed probesets (between brackets) are indicated

To gain an insight into the modulation of genes and HERV/MaLR expression, according to a well-accepted proxy of immune status in patients (i.e., HLA-DR expression on monocytes), we first performed a principal component analysis (PCA) (Fig. 3a). The PCA showed that all septic shock patients were clearly separated from HV, as demonstrated above. The low versus normal mHLA-DR groups appeared to behave differently, as shown by day 1 to day 3 orthogonal progressions for the two sub-populations. These depicted that the patients with normal mHLA-DR on day 3 tended to be closer to the HV group. In addition, when we explored the differentially expressed HERV/MaLR probesets between patients, by day of sampling and by mHLA-DR status, the first observation related to the incapacity to differentiate, on day 1, low versus normal mHLA-DR patients (Fig. 3b). A Venn diagram analysis showed that the majority of HERV/MaLR probesets appeared to be downregulated on day 3 in patients with a low, compared to normal expression of mHLA-DR (166 downregulated and 27 upregulated probesets, a total of 193 HERV/MaLR probesets, which corresponded to 162 HERV/MaLR loci).

The expression of HERV/MaLR elements was investigated and, when we compared low versus normal mHLA-DR expression on day 3, no group appeared to be enriched or depleted (Fig. 3c). Nevertheless, of the 193 differentially expressed HERV/MaLR probesets, according to mHLA-DR expression, 138 were also differentially expressed between septic shock patients on day 3 and HV. Hence, 55 HERV/MaLR probesets showed specific differential expression within septic shock patients, according to mHLA-DR expression level. Differential expression analysis of low versus normal mHLA-DR conditions also identified a downregulation of genes involved in antigen-presentation; TCR activation; differentiation of effector CD8+ T cells, such as CD3G, DPP4, and EOMES; and chemokines and cytokine receptors, such as CCL5 and IL5RA. We found that IL18R1 and CYP1B1 genes, which are involved in cytokine signaling and cell proliferation, migration and survival, HGF, IL1R2, and MERTK genes, which are involved in cell growth, apoptosis, and the TLR innate immune response, were all upregulated in low mHLA-DR samples (Fig. 3c).

We validated the relevance of the loci identified by the microarray using RT-qPCR. We selected 29 HERV/MaLR loci that were significantly differentially expressed in patients on day 3, according to the mHLA-DR status. The HERV/MaLR locus-specific RT-qPCR systems were meticulously designed and validated on genomic DNA matrix to ensure locus specificity (Additional file 6: Figure S3). Additional file 7: Figure S4 illustrates the consistency of 27 out of the 29 microarrays and RT-qPCR profiles. We found three HERV/MaLR profiles that allowed us to determine the immune status of patients on day 3 and sometimes on day 1. Approximately three quarters of the HERV/MaLR loci had a lowered expression in patients with low mHLA-DR, while the remainder conversely showed a significant increase in expression in the same patients. Interestingly, on day 1, some HERV/MaLR loci were able to stratify patients who had normal or low mHLA-DR on day 3, when no difference in mHLA-DR expression was observed on day 1. Consequently, we have identified a link between immune profiles, and the LTR retrotransposon expression.

### HERV/MaLR loci can differentiate septic shock patients according to severity status

Finally, we sought to evaluate whether the 193 HERV/MaLR probesets found to be differentially expressed between low and normal mHLA-DR patients on day 3 in the discovery phase were also expressed and able to differentiate septic shock patients in an independent and unstratified cohort subset (MIP-Rea, validation phase). The 193 identified HERV/MaLR probesets were tested on the MIP-Rea cohort, and patients were analyzed by unsupervised hierarchical clustering, according to the expression of these probesets.

Two well-separated groups of septic shock patients were highlighted (Table 2 and Additional file 8: Table S3). The first group, hereafter called cluster 1, consisted of 60 septic shock patients and the second group, called cluster 2, included 40 septic shock patients. Both groups presented significantly different SOFA severity scores on day 3 and day 6 (*P* value 0.003 and *P* value < 0.001, respectively), and all hematology criteria measured on day 3 (white cells, neutrophils, lymphocytes, platelets) were also found to be significantly different (*P* value < 0.001) (Table 2 and extended Additional file 8: Table S3). Even when outcomes were not statistically different, cluster 1 was characterized by 27% of patients presenting with an HAI and 27% that did not survive. Cluster 2 included a similar proportion of patients presenting with an HAI (25%) but with lower mortality (18%). Lastly, when we examined the expression of molecular markers, all were significantly different between the two clusters. The markers were HLA-DRA [36], S100A9 [37], CX3CR1 [38] expression on day 3, and CD74 ratio (D3/D1) [28], which are all linked to the immune system and were previously shown to be prognostic biomarkers (with either mortality or secondary infections) (Table 2 and extended Additional file 8: Table S3). Severity criteria, outcomes, and molecular markers confirmed that cluster 1 contains more severely affected patients.

These observations indicated that the HERV/MaLR probesets identified in the mHLA-DR-stratified patients (discovery phase) helped to differentiate an independent and unselected septic shock cohort (validation phase), based on the septic shock severity, outcomes, and prognostic molecular biomarkers.

## Discussion

Over the past 30 years, there has been a considerable decline in the early mortality rate during the first days after septic shock. However, 50 to 70% of the mortality still happens later, after the first week in ICU. As this may reflect a persistent immunosuppressive state [3], there is a need to identify patients who would benefit from immunostimulatory therapies [9, 10]. Although flow cytometric measurement of mHLA-DR is known to be a reliable biomarker associated with death and secondary infections in septic patients [24, 26, 39], the test format makes it difficult to implement in large, multi-centered studies and outside specialized immunology laboratories [27]. Conversely, molecular markers may be easier to introduce, as illustrated by the use of automated tests, with standardized methodologies, for pathogen detection [40]. Several transcriptomic studies have obtained promising candidate signatures to stratify septic patients [41–43]. This report suggests that additional loci, identified outside the limited scope of the exome, may help to allow an optimal stratification of patients, taking into account the inter-individual variability and the immune status in the first days after ICU admission.

Firstly, we provided an initial view of the HERV/MaLR transcriptome landscape in the whole blood of HV and septic shock patients. We highlighted that this transcriptome was modified between the two groups. Secondly, the HERV/MaLR transcriptome was differentially expressed between patients, according to their mHLA-DR expression, and we were able to stratify an independent cohort using the identified HERV/MaLR probesets, with a clear difference in severity between the two clusters of patients.

A global view of the HERV transcriptome in the whole blood was obtained using the HERV-V3 chip. We observed that approximately 6.9% of LTR retrotransposons were transcriptionally active, which is similar to the 5.6% we had previously observed, ex vivo, in a PBMCs/endotoxin tolerance model [13]. Of note, 82.4% of the expressed probesets (58,587 probesets) were shared between the whole blood and PBMCs. The differences observed between whole blood and PBMCs may be due to (i) the variability among whole blood samples, (ii) the cell type composition, such as the presence of neutrophils in the whole blood, and (iii) the stimuli released from the endothelial environment. We observed a high proportion of gamma-retroviruses, which included HERV families that potentially code for envelope proteins that comprise an immunosuppressive domain (ISD) [44]. These families included HERV-H, HERV-W, HERV-FRD, HERV-Fc2, and HERV-T [45]. Expression of the two former families was previously observed in PBMCs of healthy subjects [15, 45–48] and on B cells and monocytes of multiple sclerosis patients [49–51]. In addition, we detected the expression of poorly characterized groups, such as MER52A and PRIMA41. The promoter function of MER52A was demonstrated in HEK-293T kidney cells [52] and appears to be negatively controlled by epigenetic factors, such as methylation in stem cells or PBMCs [52]. Similar epigenetic alterations are also seen in sepsis [53, 54]. The LTRC/D of MER41 is a member of the PRIMA41 elements that have been identified as enhancers for adjacent IFN-induced genes and have been shown to be involved in the regulation of essential immune functions [21]. Overall, differential expression analysis between HV and septic shock patients confirmed that septic shock is associated with an early modulation of thousands of HERV/MaLR loci, which is in agreement with (1) in vitro LTR retrotransposons being tightly regulated to endotoxin-induced stress [13] and (2) in vivo preliminary observations in critically ill patients [12].

We showed that individual HERV/MaLR can be differentially expressed between patients, according to mHLA-DR as a proxy of the immune status. We found that 193 probesets, which represented 168 distinct HERV/MaLR loci, were differentially expressed in septic shock patients, depending on their immunosuppression status at day 3. The potential for stratification using these HERV/MaLR loci was validated on an independent cohort, with the identification of a severely affected group that had higher SOFA scores, decreased CX3CR1 expression (molecular marker associated with survival) [38], decreased CD74 and mHLA-DR expression (associated with HAI) [28, 55], and increased S100A9 expression (associated with increased risk of secondary infections and mortality) [56]. As this pilot study was designed as a proof of concept study to show that HERV transcriptome can reflect the immune status, larger studies that involve higher numbers of patients are now warranted. Some of the identified immunosuppression HERV/MaLR loci will thus be evaluated in the REALISM study [57], which aims to determine the incidence, severity, and persistence of innate and adaptive immune alterations in 550 ICU patients (sepsis, severe trauma/burn, and major surgery), compared to 180 age-matched HV. We will study whether HERV/MaLR non-conventional genes can provide added value to conventional genes [28, 38, 41, 42], in regard to the characterization of their immune status and risk prognostication.

Finally, 10% of loci in the 193 HERV/MaLR probesets, identified between septic shock patients with a low vs normal mHLA-DR expression (discovery phase), belonged to the well-annotated region of the HERV/MaLR subpart of the genome (e.g., HERV-H, HERV-L, HERV-E4.1, and MER41). Forty-eight percent and 42% corresponded to the roughly annotated HERV and MaLR repertoires, respectively. The loci were mainly found in LTRs (95%), with internal *gag pol env* genes being underrepresented (5%) in the whole blood transcriptome. Eighty-five percent of the differentially expressed HERV/MaLR loci were located within or close (≤ 1 kb) to genes, which is significantly higher than the 41% of intragenic expressed LTRs in the PBMC-related endotoxin tolerance model [13] and the 12% of intragenic expressed LTRs in cancer tissues [35]. This bias may reflect either distinct levels of transcriptional control or some putative functional implications. In addition, intragenic LTR orientation was biased towards the antisense representation, i.e., 59% of the 193 identified HERV/MaLR loci were opposed to the transcriptional orientation of the conventional gene. A similar result (65%) was found in PBMCs expressed in the endotoxin tolerance model [13]. This may reflect the capacity of the antisense HERVs to generate dsRNAs [58] that trigger cytosolic sensing and modulate innate immunity, as found in cancer, following IFN-γ exposure [20], or when using methyltransferase inhibitors that lead to a type I interferon response [19]. Conversely, 25% of the 193 identified HERV/MaLR loci presented a similar transcriptional orientation to the associated conventional gene. Although this remains to be demonstrated, they may exert cis-functions, such as promoter or polyadenylation for immunity genes, as observed in oncogenes [59, 60], with 8% and 24% being predicted as a promoter or polyA site, respectively. They may also provide trans-functions, such as being an enhancer reservoir for immunity genes, as recently illustrated by the activation of the AIM2 inflammasome through the transcription factor STAT1 binding to the MER41 loci [21]. Of note, some MER41 solo LTRs were identified and STAT1 is one of the three genes (STAT1, CCR4, and HLA-DRB1/B3) identified in this study as differentially expressed between mHLA-DR-stratified patients but not between patients and HV. Lastly, some loci may encode large proteins or small peptides that drive immunological response [44, 61–63], as exemplified by the 170369402HE41 loci that putatively encodes a truncated Env protein, which contains an ISD domain.

Our study had some limitations. Firstly, we used an experimental tool, the HERV-V3 chip, which was validated with the MAQC samples, as previously described [22]. Consequently, we deliberately chose to use a high false discovery rate (FDR at 10%) when we analyzed septic patients, as effectively used in cancer with the previous generation microarray [35, 64]. Nevertheless, the highest confidence should be in those loci that were validated by RT-qPCR. Further studies using RNA-seq could support our results by providing another unbiased view of the HERV transcriptome. Secondly, the unique stratification criteria used in the discovery cohort and the limited number of patients could be considered as restrictive. Even though mHLA-DR expression was not a gold standard for the prediction of immunosuppression status, no gold standard or clinical signs currently exist to accurately evaluate immunosuppression in the ICU. In addition, the decrease in mHLA-DR expression remains an independent predictor of bad outcomes after sepsis [7]. Indeed, HERV/MaLR identified under these conditions were able to define clusters of patients in a larger independent cohort, that were different in terms of severity criteria. To further validate these results in the ICU, the clinical relevance of some HERV/MaLR candidates, together with current conventional markers, will be evaluated using a large unbiased cohort for which the immune status will be objectively defined (REALISM project; NCT01931956). Basically, such association between HERV/MaLR expression and altered states of immunity does not evidence any direct mechanistic connection, which will require further investigations.

## Conclusions

The development of immunosuppression shortly after sepsis is now well established, and there is a growing interest in immunostimulatory treatments for these patients. However, we are still lacking the appropriate tools to precisely select the patients who may respond well to this type of treatment. In this study, we used a microarray-based approach to unveil the expression of approximately 6.9% of LTR retrotransposons, which were putatively linked to the immune response, in the whole blood. We identified a panel of 162 HERV/MaLR loci that were differentially expressed in septic shock patients stratified by mHLA-DR expression. We showed that, on an independent and unselected cohort, these loci classified patients according to the severity and distinct immune profiles. The added value of these newly identified HERV/MaLR loci should now be evaluated in a larger cohort of septic patients. If they prove to be robust, they could further serve as a stratification tool prior to immunostimulatory treatment and to monitor drug efficacy, which could contribute to the reduction of mortality in sepsis patients. More generally, we have illustrated the importance of addressing both the exome and repetitive-DNA repertoires [65] to increase our understanding of sepsis pathophysiology.

## Supplementary information

**Additional file 1 : Table S1.** The HERV transcriptome in whole blood samples (descriptive phase).

**Additional file 2 : Supplementary Methods**.

**Additional file 3 : Figure S1.** Description of HERV/MaLR transcriptome in whole blood of septic shock patients from the MIP-Rea cohort subset (descriptive phase).

**Additional file 4 : Figure S2**. Genomic, transcriptomic and functional projections of the “HERV_prototype” repertoire in the IMMUNOSEPSIS cohort subset (descriptive phase).

**Additional file 5 : Table S2.** Differential expression of HERV-V3 chip repertoires induced by immune state changes in whole blood samples (Healthy volunteers and IMMUNOSEPSIS cohort subset, discovery phase).

**Additional file 6 : Figure S3.** Selection, design and quality criteria for the locus-specific qPCR systems, illustrated with the 060400302-HERV0489uL locus.

**Additional file 7 : Figure S4.** RT-qPCR validation of 29 HERV/MaLR loci that were identified by microarray and were differentially expressed, according to themHLA-DR expression (discovery phase).

**Additional file 8 : Table S3.** MIP-Rea patient characteristics according to cluster with the 193 HERV/MaLR that were differentially expressed in the IMMUNOSEPSIS cohort subset on day 3, according to mHLA-DR expression (validation phase).

## Data Availability

The microarray expression data were filed in the NCBI Gene Expression Omnibus and are accessible via GEO accession number GSE121352. All other data related to the study are available upon request to the corresponding author. The sharing of the data was submitted for approval by the REALISM consortium steering committee, as per consortium agreement.

## Abbreviations

AUC: Area under the curve
D: Day
DEG: Differentially expressed genes
DEL: Differentially expressed loci
DNA: Desoxyribonucleic acid
FC: Fold change
FDR: False discovery rate
h: Hours
HAI: Hospital-acquired infection
HERV: Human endogenous retrovirus
HV: Healthy volunteer
ICU: Intensive care unit
IFN: Interferon
ISD: Immunosuppressive domain
LTR: Long terminal repeat
MaLR: Mammalian apparent LTR retrotransposons
mHLA-DR: Monocyte HLA-DR
PBMC: Peripheral blood mononuclear cells
PCA: Principal component analysis
REALISM: REAnimation Low Immune Status Markers
RNA: Ribonucleic acid
RT-qPCR: Reverse transcription-quantitative polymerase Chain Reaction
SOFA: Sequential Organ Failure Assessment Score

## Footnotes

1 Repertoire: within HERV-V3 microarray, the probesets are grouped into four repertoire: (1) genes, (2) HERV prototype (good annotation), (3) HERV/MaLR Dfam (lower quality annotation), and (4) other probesets (such as lncRNA)

2 Class: each HERV group belongs to different classes: class I (Gamma- and Epsilon-like), II (Beta-like), and III (Spuma-like), according to their *pol* region and phylogeny similarity

3 Group: also improperly named as “families.” HERV loci belongs to HERV groups based on sequence (nucleotide and Pol amino acid), similarity, degree of fit, and taxonomic markers

4 LTR: repeat flanking sequence of HERV that allows the regulation of their expression may have a promoter or polyadenylation signal function
