# Supplementary material for: Dynamic LTR retrotransposon transcriptome landscape in septic shock patients
Source: Crit Care. 2020 Mar 18;24:96. doi: 10.1186/s13054-020-2788-8 (PMC7081582; doi:10.1186/s13054-020-2788-8)
Supplement: Supplementary file 2 — Additional file 2 : Supplementary Methods. [file 13054_2020_2788_MOESM2_ESM.doc]

**SUPPLEMENTARY METHODS**

**LTR function analysis**

For the LTR functions analysis, we attributed LTR putative function as explained in [1]. In brief, an LTR was assigned as a Promoter (Pr) if U5 expression was three times higher than U3 signal and above the background threshold. An LTR was assigned as a polyadenylation signal (pA) if U3 expression was three times higher than U5 signal and above the background threshold. If both U3 and U5 had less than three times difference in their signals and were above background threshold the expression was assigned as readthrough (RdT). The LTR was classed as silent if both the U3 and U5 signals were below the background threshold.

**Primer Design, Real-Time PCR and data analysis**

Were possible, PCR primers were designed against overlapping probes that allowed their detection on the chip. Design of locus-specific primer pairs was based on the HERV-V3 microarray analysis, using Primer3 and the NCBI Primer-BLAST software ([www.ncbi.nlm.nih.gov/tools/primer-blast](http://www.ncbi.nlm.nih.gov/tools/primer-blast)). Designs were checked *in silico* at UCSC ([https://genome.ucsc.edu](https://genome.ucsc.edu/)). The HPLC-purified primers were manufactured by Eurogentec. Specificity and sensitivity of the systems were evaluated on 1 ng of human genomic DNA (Promega) by varying the annealing temperature (Tm) from 52°C to 60°C. The amplification cycles were followed by High Resolution Melting (HRM), using the Rotor Gene Q (Qiagen), gel electrophoresis analysis on the Bioanalyzer 2100 (Agilent) and Sanger sequencing (GATC Biotech). Systems were validated if primer pairs followed three criteria: (i) only one HRM peak, (ii) fragment size corresponding to the expected product and (iii) Sanger sequencing data gave a match to the targeted locus sequence. Primer pairs, along with an illustration of the experimental validation scheme, were summarized in Additional file 6: Figure S3. For Real‑Time PCR, a single annealing temperature (56°C) was selected for all validated HERVs/MaLRs systems. All criteria were validated for all systems at this temperature, which allowed homogenization of the experiment. The 24 primer pairs that satisfied our acceptance criteria were used to amplify the original RNA samples used for the microarray experiments. The mRNA expression levels were quantified using RT-qPCR. Total RNA (100ng) was DNAse-treated and reverse transcribed using the QuantiTec Reverse Transcription Kit (Qiagen). Reverse-transcriptase-free reactions were carried out to verify the absence of contaminating genomic DNA using the TaqMan Gene Expression Assay Human 18S system Hs03003631-g1 and TaqMan Universal PCR kit (ThermoFisher). For the HERVs/MaLRs systems, SYBR green experiments were set up using the Type-it HRM PCR kit, in 20 µL final reaction volumes, with 0.7 µM primers and a 10-fold cDNA dilution (2 ng RNA equivalent). The PCR amplifications were carried out in strip tubes that were closed by caps (Qiagen). The cDNA amplifications were performed using the Rotor Gene Q as follows: a 5 min denaturation step at 95°C, followed by 40 cycles (95°C for 10s, Tm for 30s, 72°C for 10s) and HRM analysis (from 65°C to 95°C, 0.1°C increments every 2s). All reactions were performed in duplicate. Expression of housekeeping genes; Peptidylprolyl Isomerase B (PPIB) and Ribosomal Protein Lateral Stalk Subunit 0 (RPLP0), was monitored for normalization and other gene expression was investigated using TaqMan Universal PCR Master Mix and TaqMan Gene Expression Assay Human: PPIB (Hs00168719_m1), RPLP0 (Hs00420895_gH) (ThermoFisher). The PCR reactions were performed using the Rotor Gene Q, in strip tubes with 20 µL final reaction volumes. Primer concentration, mix concentration and amplification program were determined in accordance with the manufacturer’s instructions (Thermofisher). The fold change (FC) was determined using the 2^-ΔΔCt^ method. The first ΔCT is the difference in threshold cycle between the target and the geometric mean of PPIB and RPLP0 genes. The ΔΔCT is the difference in ΔCT between the target and the healthy volunteer samples. The final value of the healthy volunteer samples was arbitrarily set to one and other values were scaled up in order to provide a final relative differential expression.

1. Mommert M, Tabone O, Oriol G, Cerrato E, Guichard A, Naville M, Fournier P, Volff JN, Pachot A, Monneret *G et* al**: LTR-retrotransposon transcriptome modulation in response to endotoxin-induced stress in PBM**Cs*. BMC genomic*s 2018**,** 19(1):522.
