# Supplementary material for: Dynamic LTR retrotransposon transcriptome landscape in septic shock patients
Source: Crit Care. 2020 Mar 18;24:96. doi: 10.1186/s13054-020-2788-8 (PMC7081582; doi:10.1186/s13054-020-2788-8)
Supplement: Supplementary file 3 — Additional file 3 : Figure S1. Description of HERV/MaLR transcriptome in whole blood of septic shock patients from the MIP-Rea cohort subset (descriptive phase). [file 13054_2020_2788_MOESM3_ESM.ppt]

## Slide 1
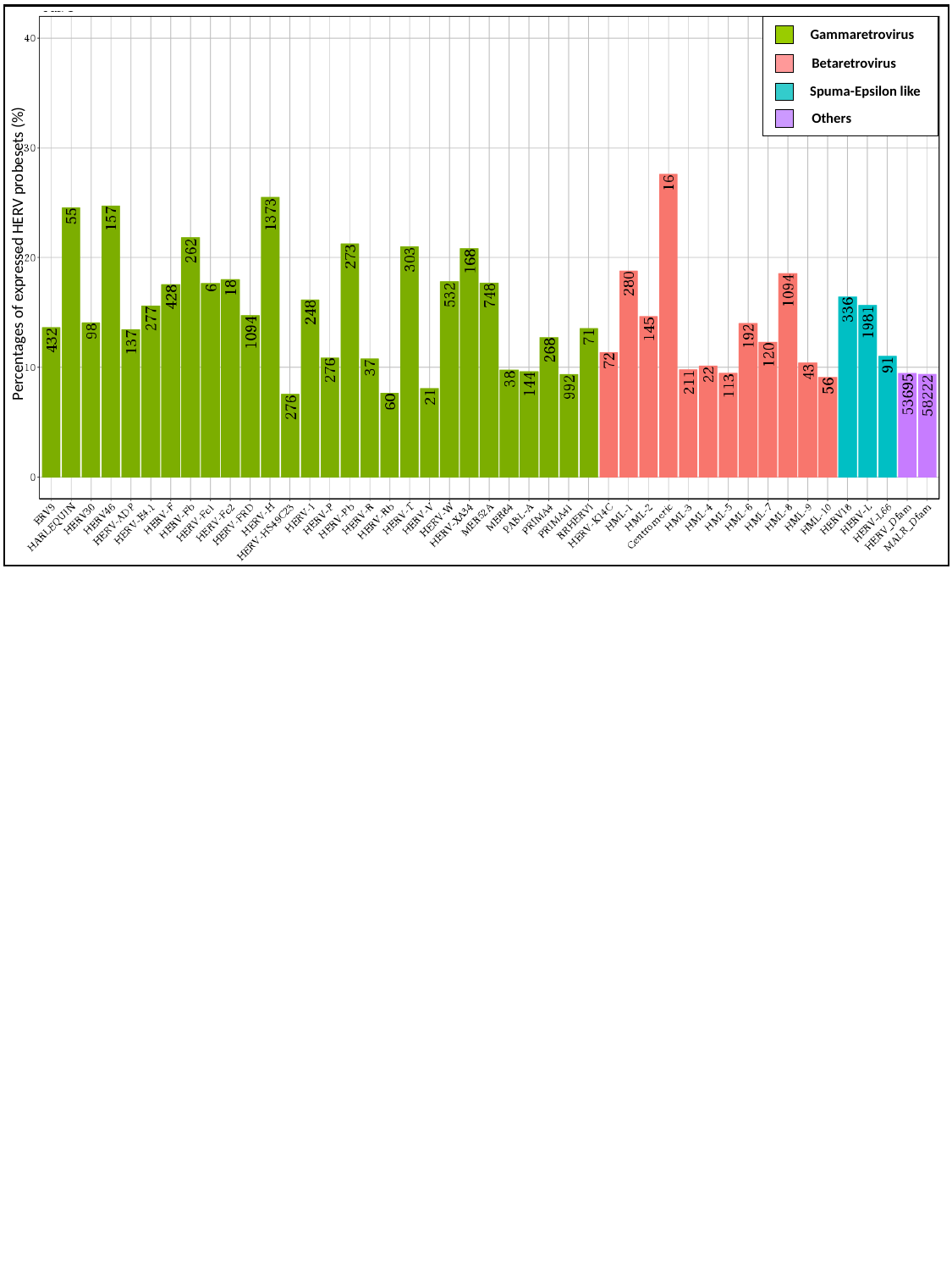

Gammaretrovirus
Betaretrovirus
Spuma-Epsilon like
Others
Percentages of expressed HERV probesets (%)
