# Supplementary material for: Dynamic LTR retrotransposon transcriptome landscape in septic shock patients
Source: Crit Care. 2020 Mar 18;24:96. doi: 10.1186/s13054-020-2788-8 (PMC7081582; doi:10.1186/s13054-020-2788-8)
Supplement: Supplementary file 4 — Additional file 4 : Figure S2. Genomic, transcriptomic and functional projections of the “HERV_prototype” repertoire in the IMMUNOSEPSIS cohort subset (descriptive phase). [file 13054_2020_2788_MOESM4_ESM.ppt]

## Slide 1
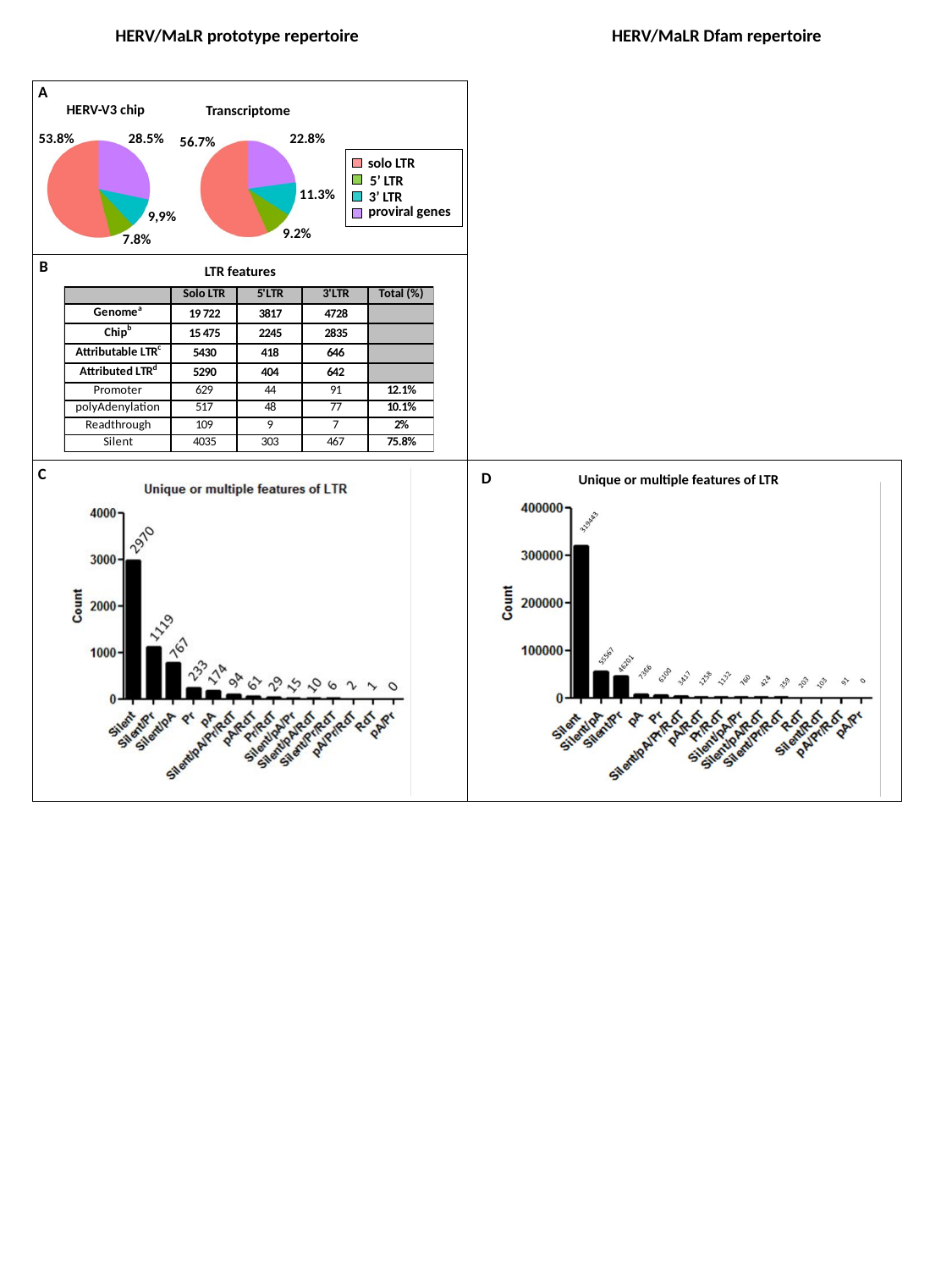

HERV/MaLR prototype repertoire
HERV/MaLR Dfam repertoire
A
HERV-V3 chip
Transcriptome
28.5%
22.8%
53.8%
56.7%
solo LTR
5’ LTR
11.3%
3’ LTR
proviral genes
9,9%
9.2%
7.8%
B
LTR features
C
D
Unique or multiple features of LTR
