# Supplementary material for: Dynamic LTR retrotransposon transcriptome landscape in septic shock patients
Source: Crit Care. 2020 Mar 18;24:96. doi: 10.1186/s13054-020-2788-8 (PMC7081582; doi:10.1186/s13054-020-2788-8)
Supplement: Supplementary file 6 — Additional file 6 : Figure S3. Selection, design and quality criteria for the locus-specific qPCR systems, illustrated with the 060400302-HERV0489uL locus. [file 13054_2020_2788_MOESM6_ESM.ppt]

## Slide 1
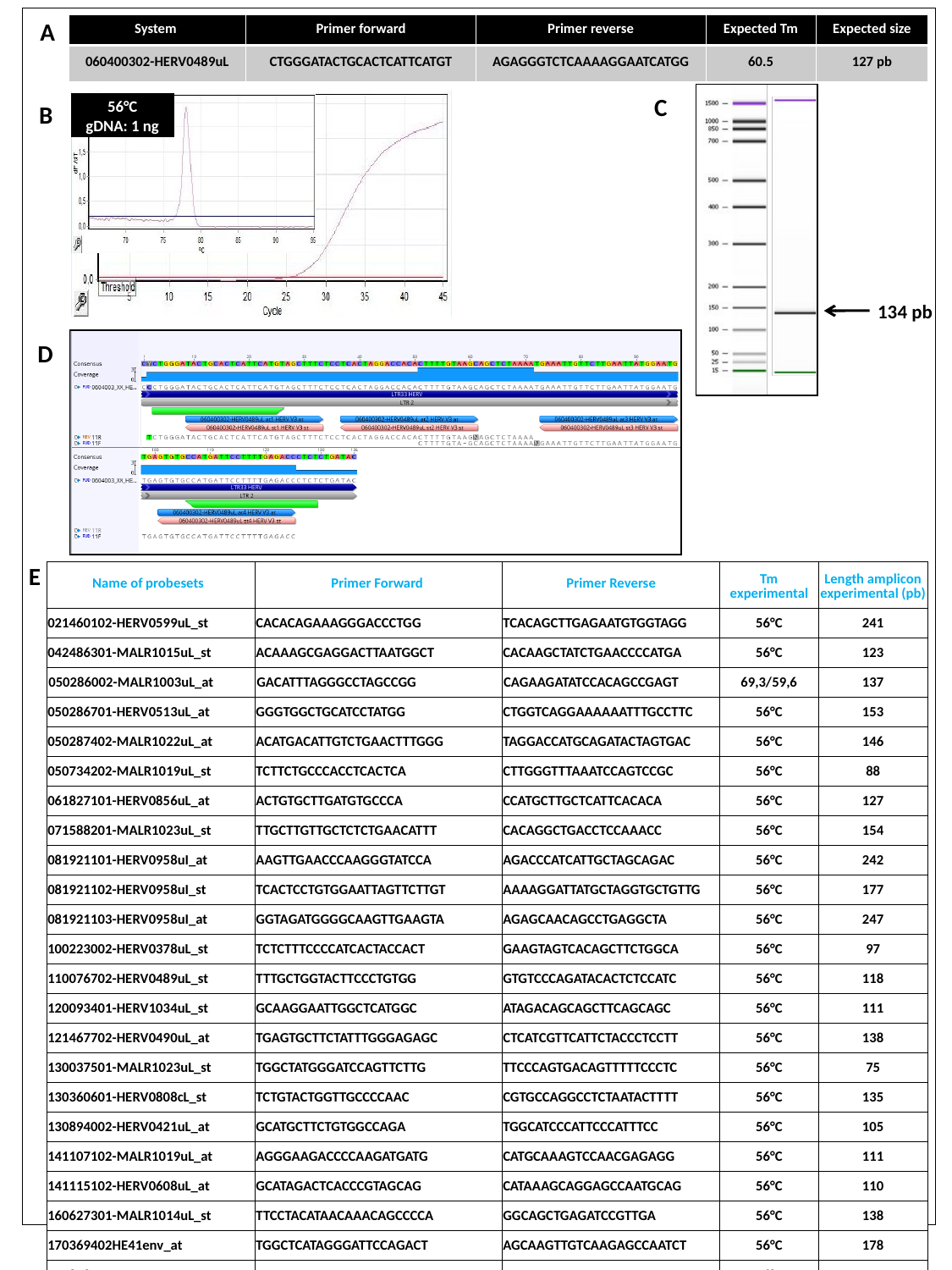

A
| System | Primer forward | Primer reverse | Expected Tm | Expected size |
| --- | --- | --- | --- | --- |
| 060400302-HERV0489uL | CTGGGATACTGCACTCATTCATGT | AGAGGGTCTCAAAAGGAATCATGG | 60.5 | 127 pb |
C
B
56°C
gDNA: 1 ng
134 pb
D
E
| Name of probesets | Primer Forward | Primer Reverse | Tm experimental | Length amplicon experimental (pb) |
| --- | --- | --- | --- | --- |
| 021460102-HERV0599uL\_st | CACACAGAAAGGGACCCTGG | TCACAGCTTGAGAATGTGGTAGG | 56°C | 241 |
| 042486301-MALR1015uL\_st | ACAAAGCGAGGACTTAATGGCT | CACAAGCTATCTGAACCCCATGA | 56°C | 123 |
| 050286002-MALR1003uL\_at | GACATTTAGGGCCTAGCCGG | CAGAAGATATCCACAGCCGAGT | 69,3/59,6 | 137 |
| 050286701-HERV0513uL\_at | GGGTGGCTGCATCCTATGG | CTGGTCAGGAAAAAATTTGCCTTC | 56°C | 153 |
| 050287402-MALR1022uL\_at | ACATGACATTGTCTGAACTTTGGG | TAGGACCATGCAGATACTAGTGAC | 56°C | 146 |
| 050734202-MALR1019uL\_st | TCTTCTGCCCACCTCACTCA | CTTGGGTTTAAATCCAGTCCGC | 56°C | 88 |
| 061827101-HERV0856uL\_at | ACTGTGCTTGATGTGCCCA | CCATGCTTGCTCATTCACACA | 56°C | 127 |
| 071588201-MALR1023uL\_st | TTGCTTGTTGCTCTCTGAACATTT | CACAGGCTGACCTCCAAACC | 56°C | 154 |
| 081921101-HERV0958uI\_at | AAGTTGAACCCAAGGGTATCCA | AGACCCATCATTGCTAGCAGAC | 56°C | 242 |
| 081921102-HERV0958uI\_st | TCACTCCTGTGGAATTAGTTCTTGT | AAAAGGATTATGCTAGGTGCTGTTG | 56°C | 177 |
| 081921103-HERV0958uI\_at | GGTAGATGGGGCAAGTTGAAGTA | AGAGCAACAGCCTGAGGCTA | 56°C | 247 |
| 100223002-HERV0378uL\_st | TCTCTTTCCCCATCACTACCACT | GAAGTAGTCACAGCTTCTGGCA | 56°C | 97 |
| 110076702-HERV0489uL\_st | TTTGCTGGTACTTCCCTGTGG | GTGTCCCAGATACACTCTCCATC | 56°C | 118 |
| 120093401-HERV1034uL\_st | GCAAGGAATTGGCTCATGGC | ATAGACAGCAGCTTCAGCAGC | 56°C | 111 |
| 121467702-HERV0490uL\_at | TGAGTGCTTCTATTTGGGAGAGC | CTCATCGTTCATTCTACCCTCCTT | 56°C | 138 |
| 130037501-MALR1023uL\_st | TGGCTATGGGATCCAGTTCTTG | TTCCCAGTGACAGTTTTTCCCTC | 56°C | 75 |
| 130360601-HERV0808cL\_st | TCTGTACTGGTTGCCCCAAC | CGTGCCAGGCCTCTAATACTTTT | 56°C | 135 |
| 130894002-HERV0421uL\_at | GCATGCTTCTGTGGCCAGA | TGGCATCCCATTCCCATTTCC | 56°C | 105 |
| 141107102-MALR1019uL\_at | AGGGAAGACCCCAAGATGATG | CATGCAAAGTCCAACGAGAGG | 56°C | 111 |
| 141115102-HERV0608uL\_at | GCATAGACTCACCCGTAGCAG | CATAAAGCAGGAGCCAATGCAG | 56°C | 110 |
| 160627301-MALR1014uL\_st | TTCCTACATAACAAACAGCCCCA | GGCAGCTGAGATCCGTTGA | 56°C | 138 |
| 170369402HE41env\_at | TGGCTCATAGGGATTCCAGACT | AGCAAGTTGTCAAGAGCCAATCT | 56°C | 178 |
| 170828901-HERV0770cL\_at | GCCTGAGCGTTTTTATGCTGG | AGTACACAGGTCTTGCCGAGA | 56°C | 119 |
| 190148802-MALR1127uL\_at | GACTGGTTTTGAACTCCTAGGCT | CACCCCTCCCAAATGCATATGT | 56°C | 183 |
| 190478501-MALR1003cL\_st | GCATGTTGGTTTGTGTCTTGGT | CAGCGTGCAGTGGTGTGA | 56°C | 116 |
| 220246901-HERV0889uL\_at | AAATGAACACCTGGGCAAACAA | TGCCTAACGTAAGCACCCATC | 56°C | 126 |
| 220247002-HERV0797uL\_at | GCCAGAGAGGCATAATGAAGCA | GATTCTAAGCCTCCCCCTCATTT | 56°C | 129 |
| | | | | |
| 032622601MR41sLU5p\_st | TTGCAGGAAAGGGGTGCAG | TTACTCTATGGACTCGCCCTGA | 56°C | 96 |
| 100468701-MALR0820cL\_st | ATAAAGGAATACCTGAGGCTGGG | CACTGAGCTCGGCCTCTTC | 56°C | 221 |
