# Supplementary material for: Dynamic LTR retrotransposon transcriptome landscape in septic shock patients
Source: Crit Care. 2020 Mar 18;24:96. doi: 10.1186/s13054-020-2788-8 (PMC7081582; doi:10.1186/s13054-020-2788-8)
Supplement: Supplementary file 7 — Additional file 7 : Figure S4. RT-qPCR validation of 29 HERV/MaLR loci that were identified by microarray and were differentially expressed, according to themHLA-DR expression (discovery phase). [file 13054_2020_2788_MOESM7_ESM.ppt]

## Slide 1
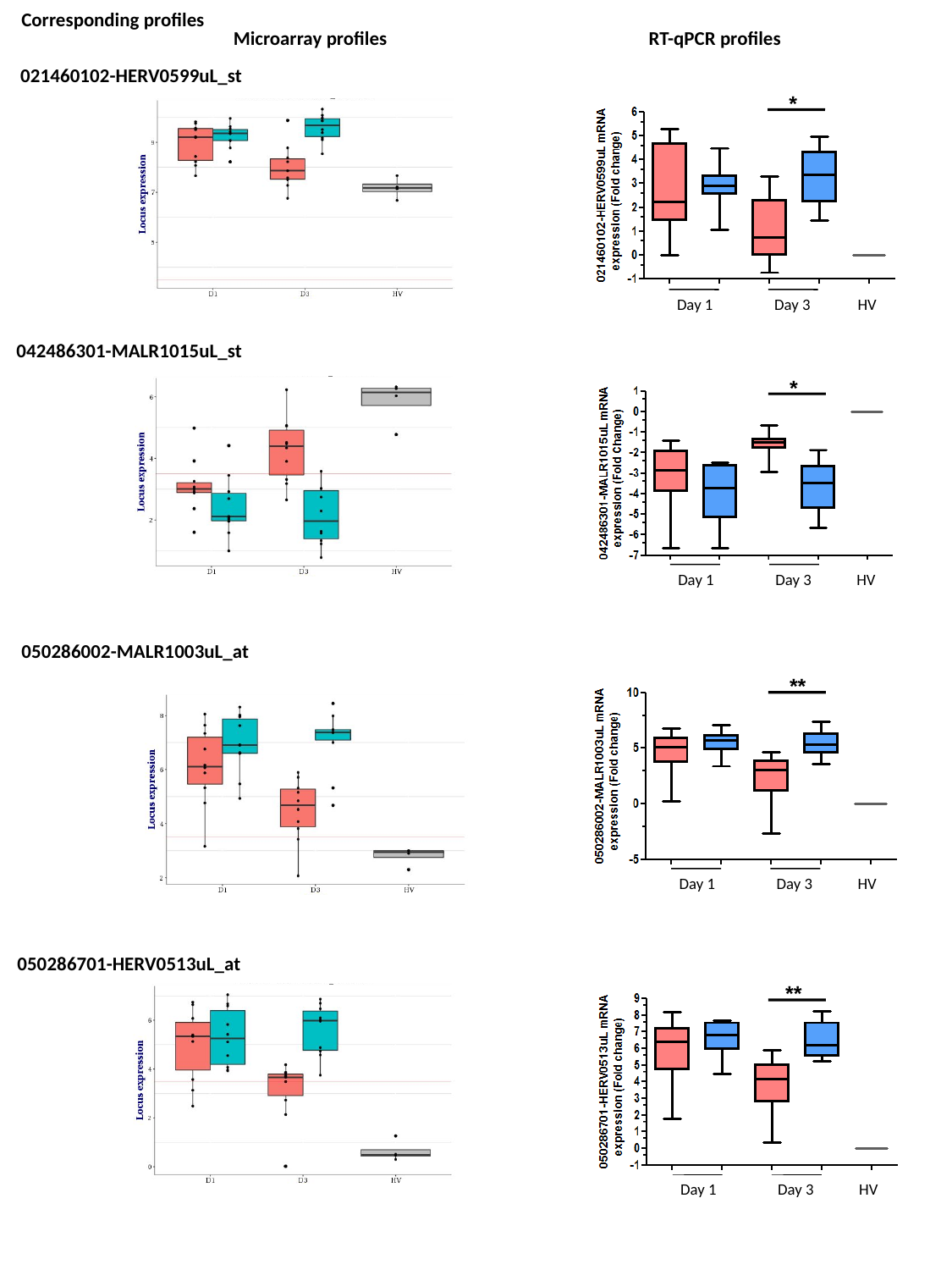

Corresponding profiles
Microarray profiles
RT-qPCR profiles
021460102-HERV0599uL_st
*
Day 1
Day 3
HV
042486301-MALR1015uL_st
*
Day 1
Day 3
HV
050286002-MALR1003uL_at
**
Day 1
Day 3
HV
050286701-HERV0513uL_at
**
Day 1
Day 3
HV

## Slide 2
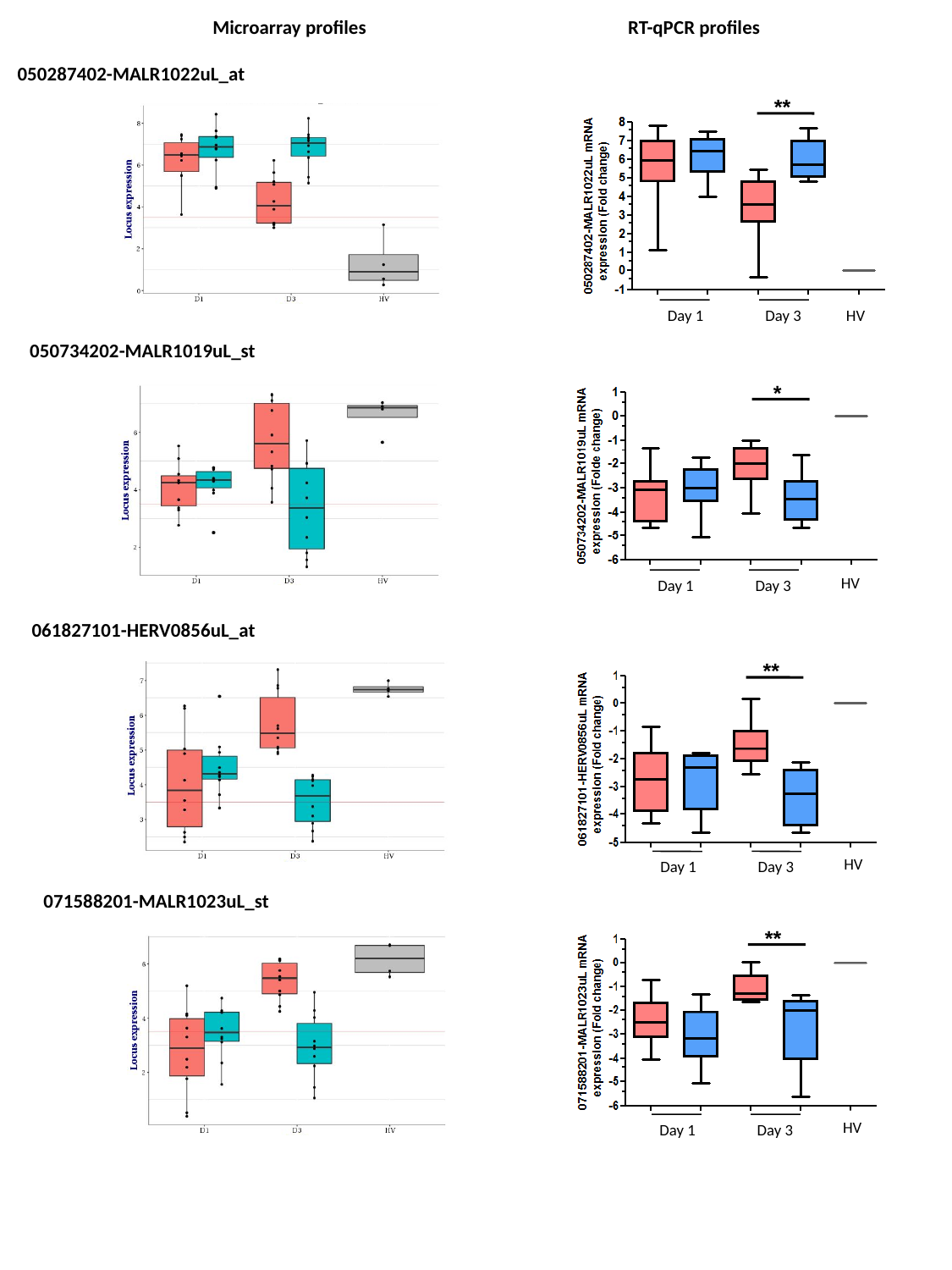

Microarray profiles
RT-qPCR profiles
050287402-MALR1022uL_at
**
Day 1
Day 3
HV
050734202-MALR1019uL_st
*
HV
Day 1
Day 3
061827101-HERV0856uL_at
**
HV
Day 1
Day 3
071588201-MALR1023uL_st
**
HV
Day 1
Day 3

## Slide 3
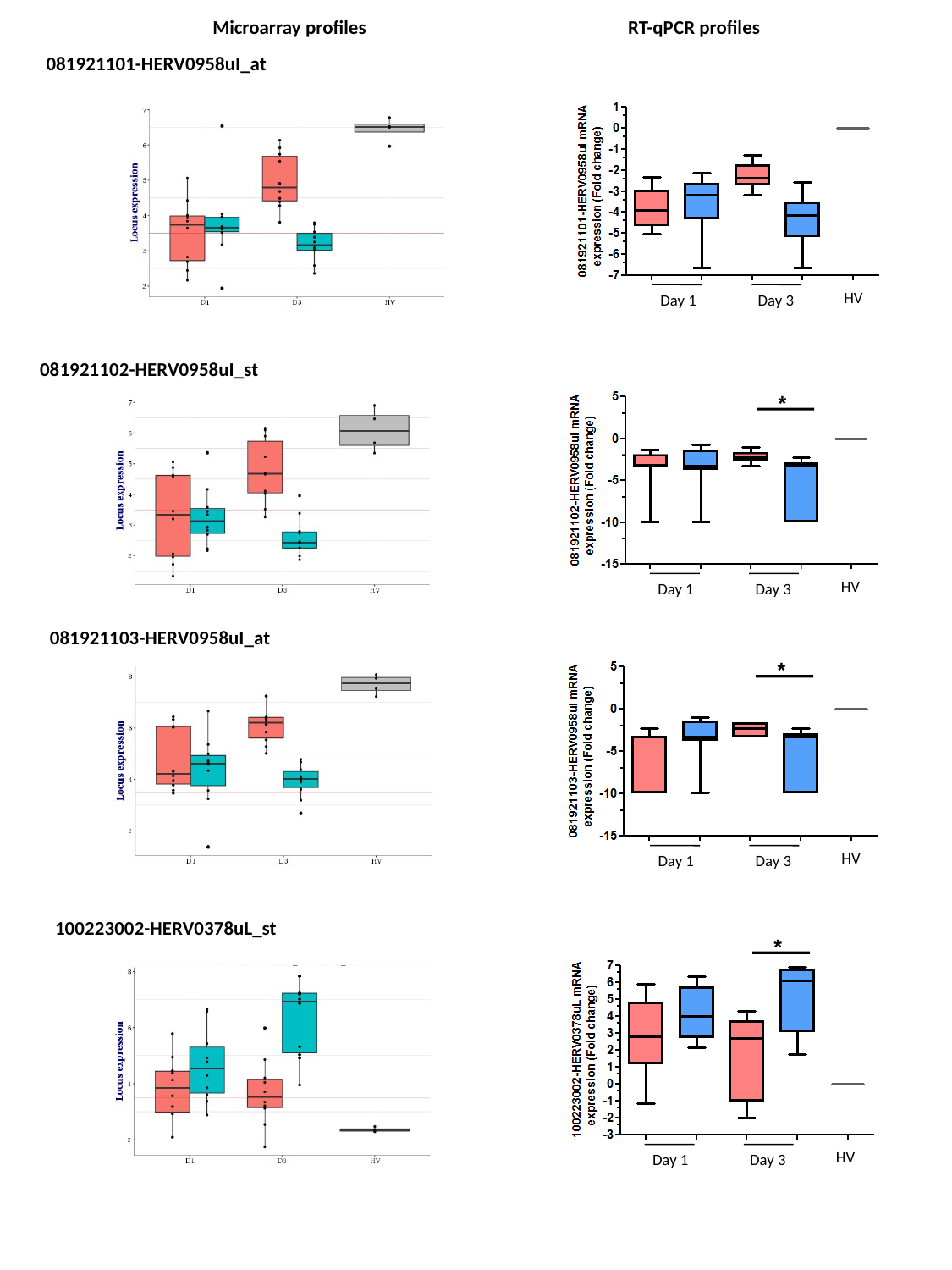

Microarray profiles
RT-qPCR profiles
081921101-HERV0958uI_at
HV
Day 1
Day 3
081921102-HERV0958uI_st
*
HV
Day 1
Day 3
081921103-HERV0958uI_at
*
HV
Day 1
Day 3
100223002-HERV0378uL_st
*
HV
Day 1
Day 3

## Slide 4
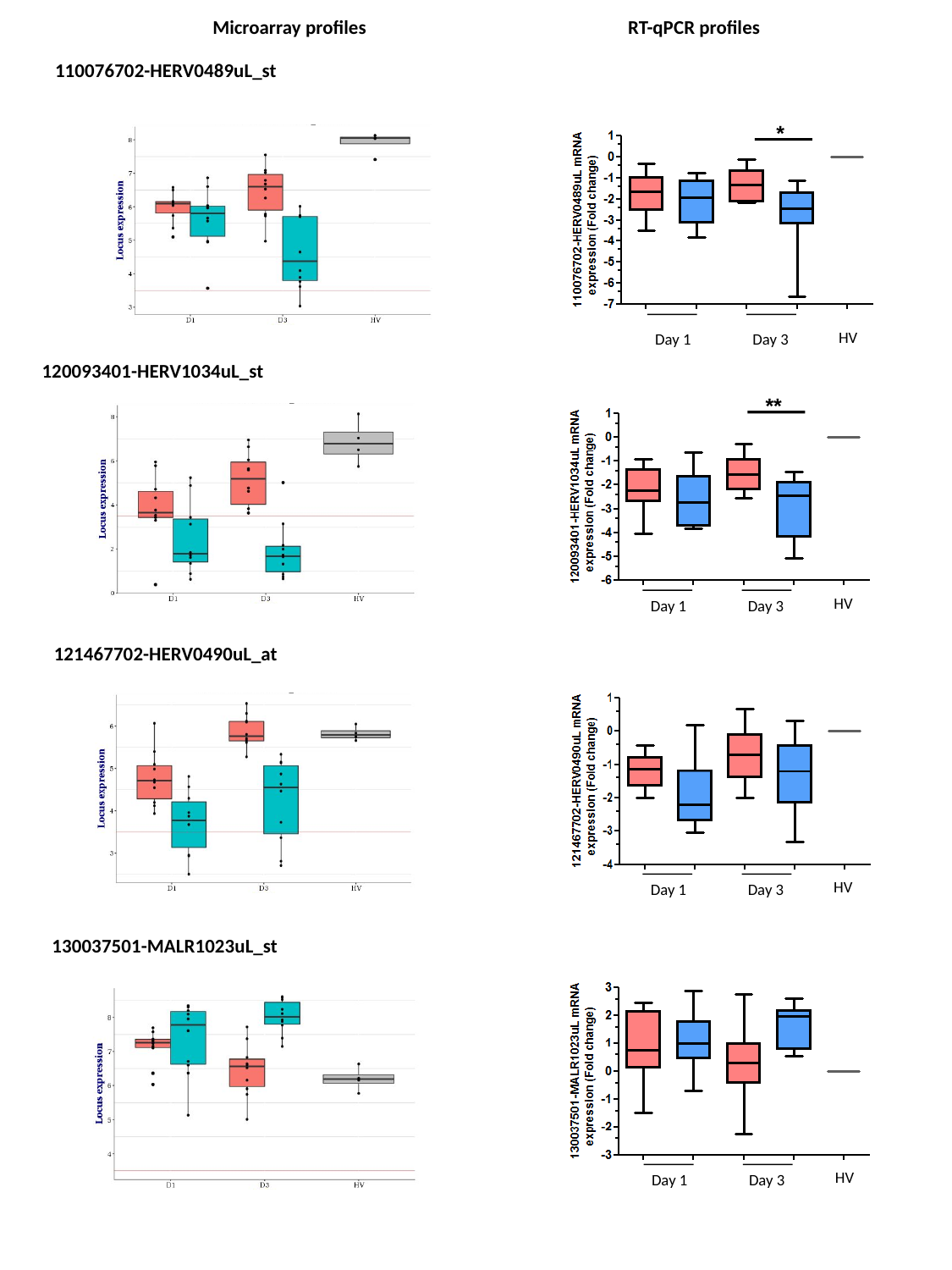

Microarray profiles
RT-qPCR profiles
110076702-HERV0489uL_st
*
HV
Day 1
Day 3
120093401-HERV1034uL_st
**
HV
Day 1
Day 3
121467702-HERV0490uL_at
HV
Day 1
Day 3
130037501-MALR1023uL_st
HV
Day 1
Day 3

## Slide 5
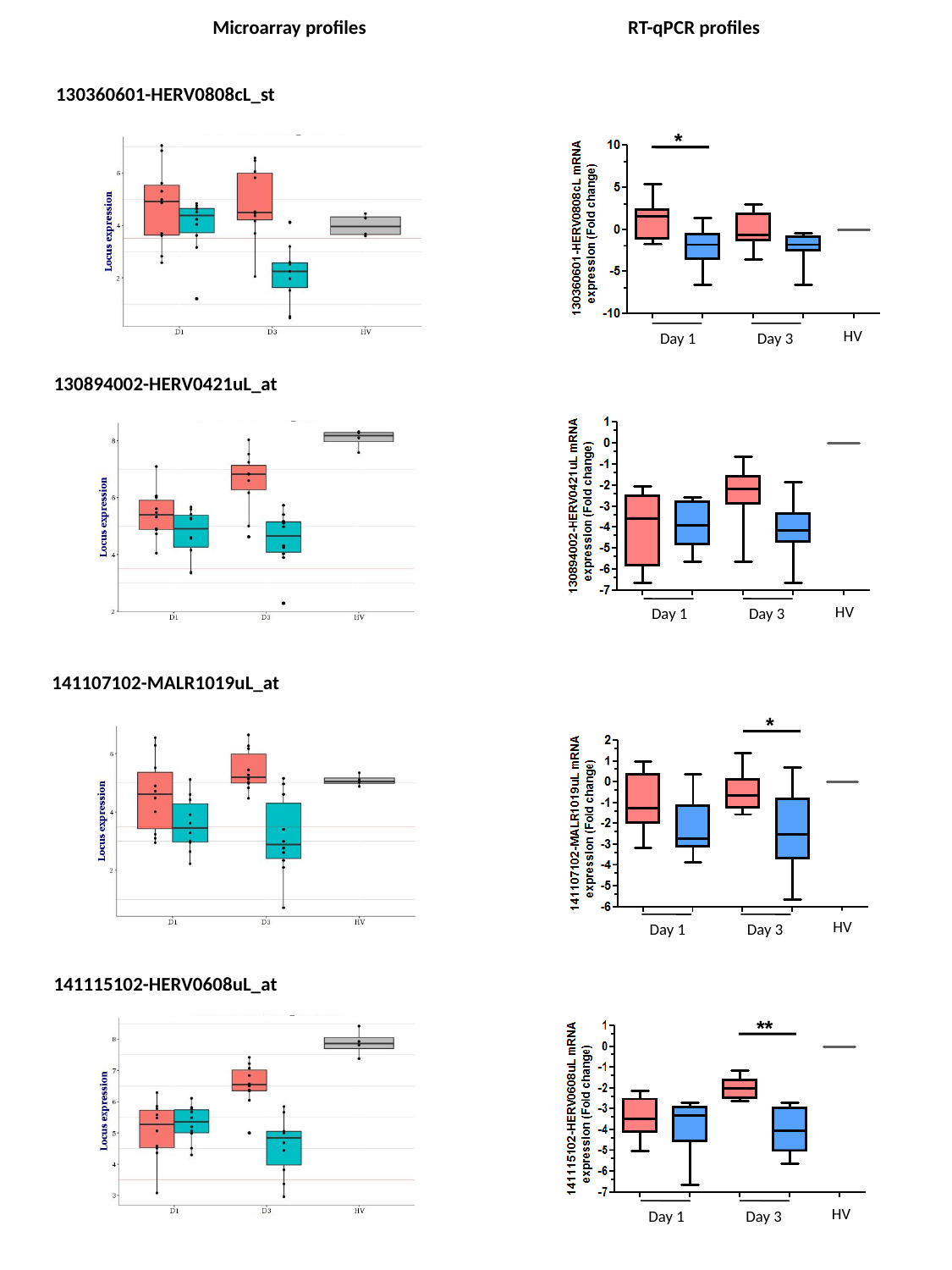

Microarray profiles
RT-qPCR profiles
130360601-HERV0808cL_st
*
HV
Day 1
Day 3
130894002-HERV0421uL_at
HV
Day 1
Day 3
141107102-MALR1019uL_at
*
HV
Day 1
Day 3
141115102-HERV0608uL_at
**
HV
Day 1
Day 3

## Slide 6
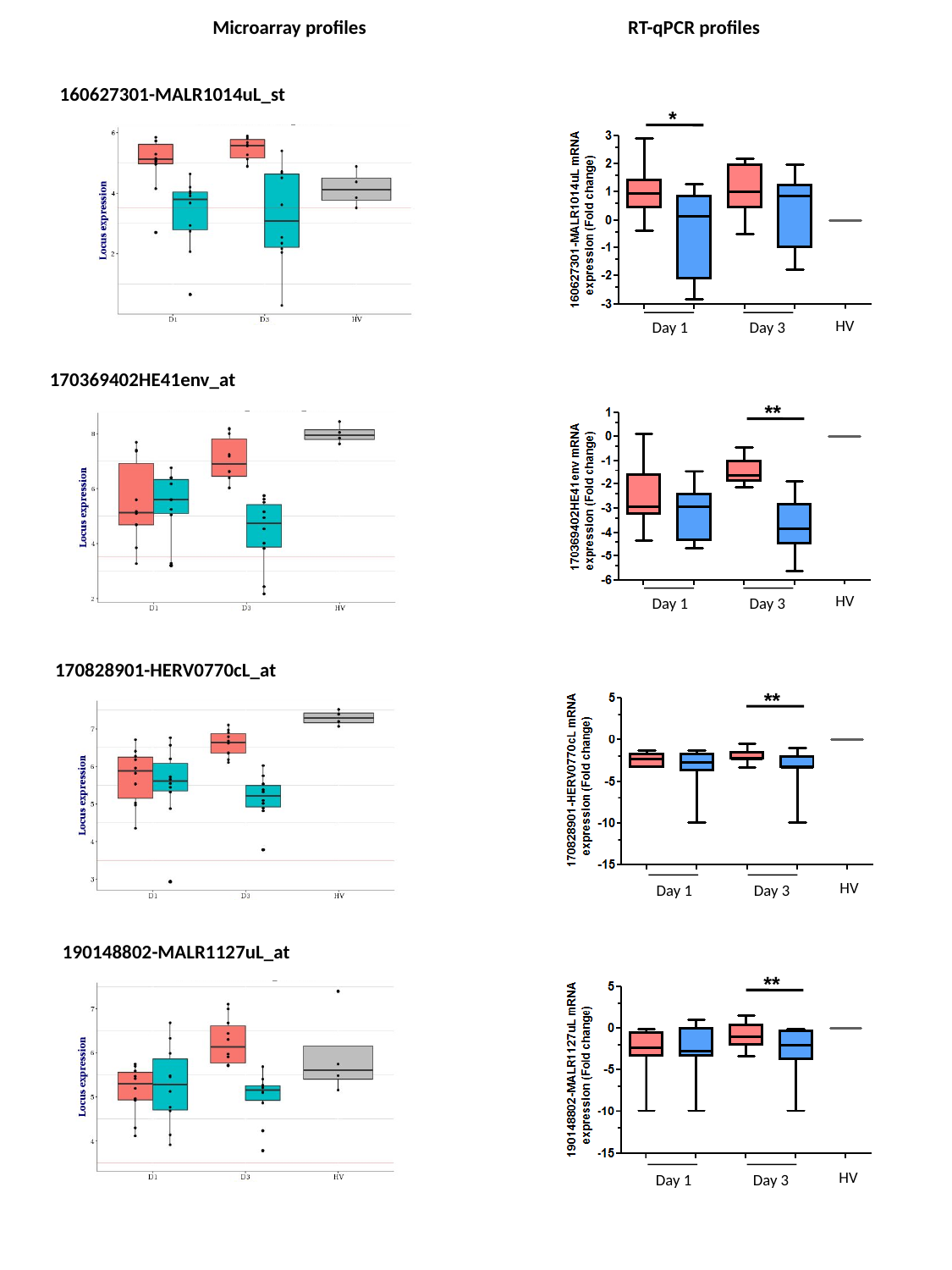

Microarray profiles
RT-qPCR profiles
160627301-MALR1014uL_st
*
HV
Day 1
Day 3
170369402HE41env_at
**
HV
Day 1
Day 3
170828901-HERV0770cL_at
**
HV
Day 1
Day 3
190148802-MALR1127uL_at
**
HV
Day 1
Day 3

## Slide 7
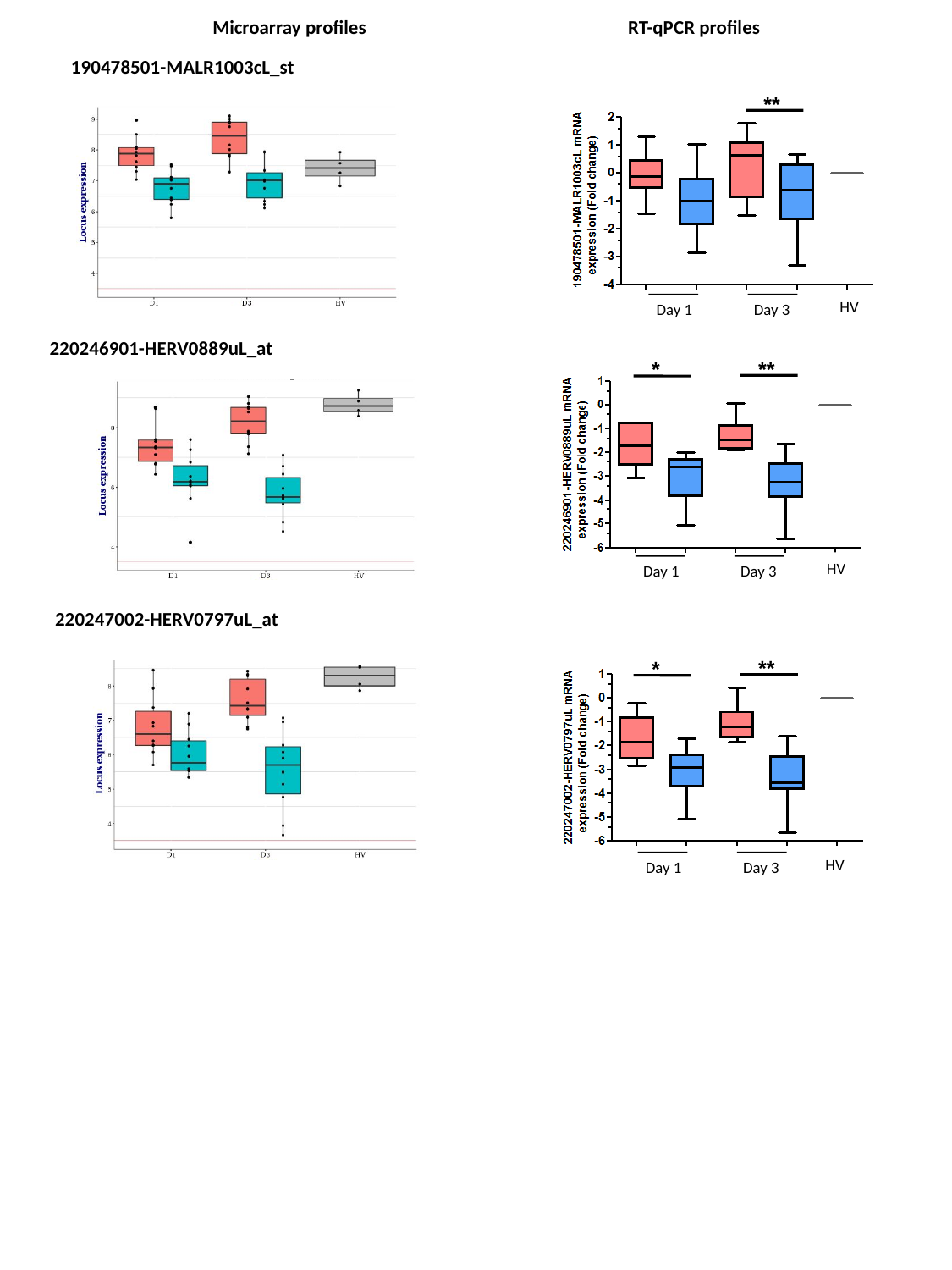

Microarray profiles
RT-qPCR profiles
190478501-MALR1003cL_st
**
HV
Day 1
Day 3
220246901-HERV0889uL_at
**
*
HV
Day 1
Day 3
220247002-HERV0797uL_at
**
*
HV
Day 1
Day 3

## Slide 8
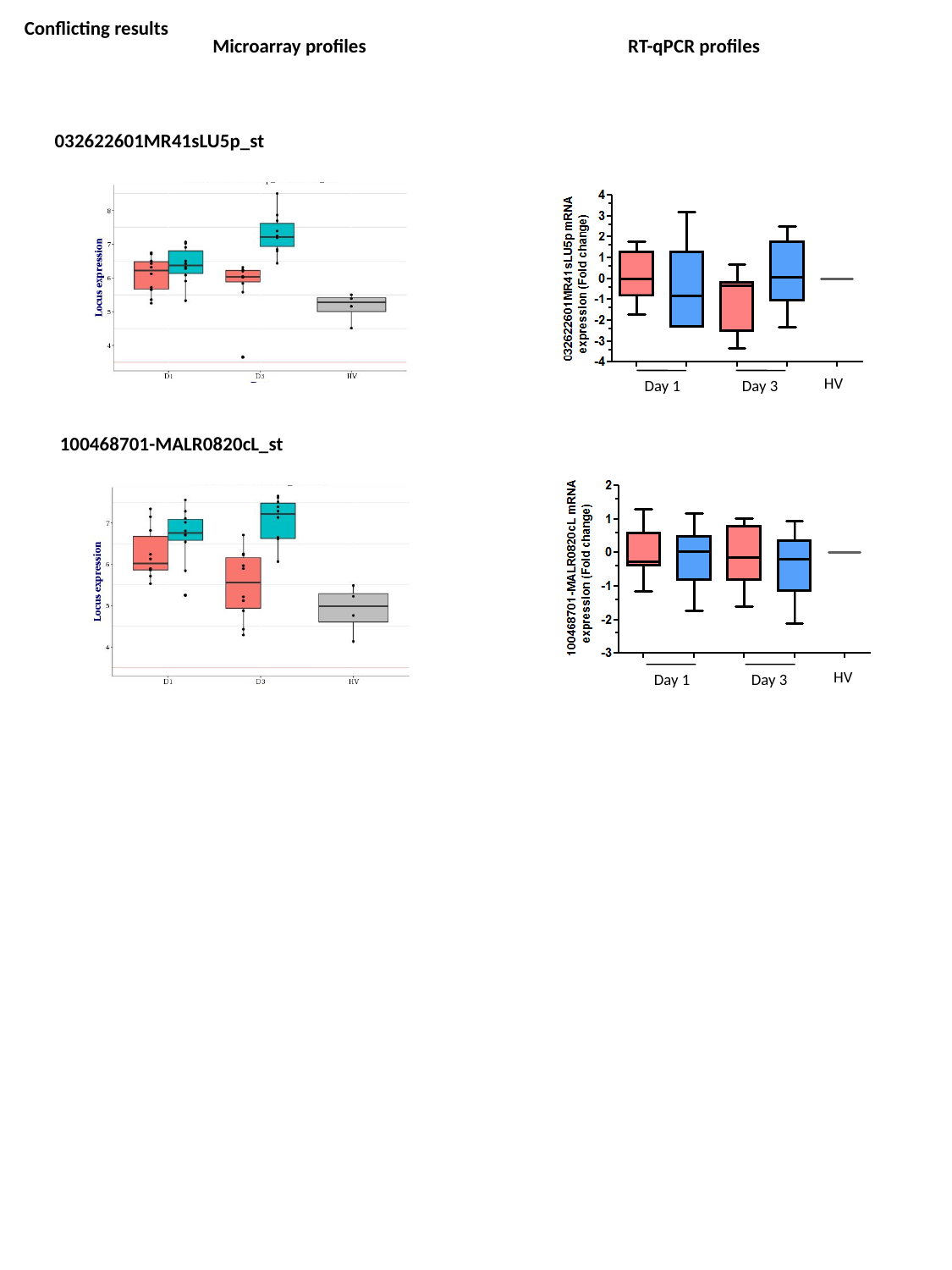

Conflicting results
Microarray profiles
RT-qPCR profiles
032622601MR41sLU5p_st
HV
Day 1
Day 3
100468701-MALR0820cL_st
HV
Day 1
Day 3
